# Identification of a prognostic signature in colorectal cancer using combinatorial algorithm‐driven analysis

**DOI:** 10.1002/cjp2.258

**Published:** 2022-01-18

**Authors:** Abdo Alnabulsi, Tiehui Wang, Wei Pang, Marius Ionescu, Stephanie G Craig, Matthew P Humphries, Kris McCombe, Manuel Salto Tellez, Ayham Alnabulsi, Graeme I Murray

**Affiliations:** ^1^ Pathology, School of Medicine, Medical Sciences and Nutrition University of Aberdeen Aberdeen UK; ^2^ AiBIOLOGICS Dublin Ireland; ^3^ School of Biological Sciences University of Aberdeen Aberdeen UK; ^4^ School of Mathematical & Computer Sciences Heriot‐Watt University Edinburgh UK; ^5^ Precision Medicine Centre, Patrick G Johnson Centre for Cancer Research Queen's University Belfast Belfast UK; ^6^ Vertebrate Antibodies Ltd, Zoology Building University of Aberdeen Aberdeen UK

**Keywords:** biomarker, colorectal cancer, combinatorial analysis, combinatorial algorithm, immunohistochemistry, prognosis, tissue microarray

## Abstract

Colorectal carcinoma is one of the most common types of malignancy and a leading cause of cancer‐related death. Although clinicopathological parameters provide invaluable prognostic information, the accuracy of prognosis can be improved by using molecular biomarker signatures. Using a large dataset of immunohistochemistry‐based biomarkers (*n* = 66), this study has developed an effective methodology for identifying optimal biomarker combinations as a prognostic tool. Biomarkers were screened and assigned to related subsets before being analysed using an iterative algorithm customised for evaluating combinatorial interactions between biomarkers based on their combined statistical power. A signature consisting of six biomarkers was identified as the best combination in terms of prognostic power. The combination of biomarkers (STAT1, UCP1, p‐cofilin, LIMK2, FOXP3, and ICOS) was significantly associated with overall survival when computed as a linear variable (*χ* ^2^ = 53.183, *p* < 0.001) and as a cluster variable (*χ* ^2^ = 67.625, *p* < 0.001). This signature was also significantly independent of age, extramural vascular invasion, tumour stage, and lymph node metastasis (Wald = 32.898, *p* < 0.001). Assessment of the results in an external cohort showed that the signature was significantly associated with prognosis (*χ* ^2^ = 14.217, *p* = 0.007). This study developed and optimised an innovative discovery approach which could be adapted for the discovery of biomarkers and molecular interactions in a range of biological and clinical studies. Furthermore, this study identified a protein signature that can be utilised as an independent prognostic method and for potential therapeutic interventions.

## Introduction

Colorectal cancer is a common malignancy with a relatively high mortality rate and a significant negative impact on the quality of life of survivors [1]. Although mortality rates of colorectal cancer have been declining in developed countries as a result of significant development in health care, deaths attributed to colorectal cancer, already one of the highest contributors to cancer‐related mortalities, are expected to continue rising due to ageing and diet [2, 3].

The main obstacle to better survival rates is the molecular heterogeneity of colorectal cancer which is reflected clinically through variations in tumour progression, prognosis, and response to treatment [4]. The profiling of large sets of genes/proteins and the identification of molecular signatures are needed to subtype colorectal cancer and manage patients accordingly [5]. The molecular analysis of colorectal cancer improves our understanding of tumourigenesis and uncovers novel pathways, which can be utilised in prognosis, screening, monitoring, and therapeutic interventions [6, 7].

Because of the complexity and the heterogeneity of colorectal cancer, multiple biomarkers are needed for the necessary prognostic power to accurately subtype this disease. However, discovery of novel biomarkers with high‐order combinatorial interactions is extremely challenging due to the combinatorial explosion (the size of genomic/proteomic data and arbitrariness of combinations) and constraints of multiple hypothesis testing for significance evaluation [8].

This study has developed an effective method which minimises the computational complexity to identify the optimal prognostic combination from a large number of biomarkers. This method comprises a screening process to eliminate non‐relevant targets, followed by a grouping stage whereby the remaining biomarkers are divided into smaller subsets. Each subset is then incorporated into an iterative algorithm which generates and evaluates all biomarker combinations. Following this method, the study has identified a biomarker signature with a significant prognostic power to predict the overall survival of patients independent of established prognostic parameters. Furthermore, important biological pathways in colorectal cancer were revealed by analysing associations, expression patterns, and functional interactions of these biomarkers.

## Materials and methods

### Discovery patient cohort

To assess the expression of biomarker targets, a large and well‐characterised patient cohort of primary colorectal cancers was used. The cohort was retrospectively acquired from the Grampian Biorepository (www.biorepository.nhsgrampian.org), and only included patients who had undergone surgery with curative intent for primary colorectal cancers between 1994 and 2009, at Aberdeen Royal Infirmary‐NHS Grampian (Aberdeen, UK). This study also followed the Reporting Recommendations for Tumour Marker Prognostic Studies (REMARK) guidelines [9] (checklist included in supplementary material).

Only patients with Union for International Cancer Control (UICC) TNM stage I, stage II, or stage III were included in the study (*n* = 650). Patients with histological evidence of distant metastasis at diagnosis or those who had received neoadjuvant chemotherapy and/or radiotherapy were not included. The histopathological reporting of the resection specimens was performed following the relevant guidelines from the Royal College of Pathologists UK for the histopathological reporting of colorectal cancer excision specimens which incorporated TNM version 5. Further details of the histopathological processing of tissue specimens are outlined in Supplementary materials and methods.

The primary endpoint was overall survival which was defined as the period from 28 days after the date of surgery to the date of death from any cause. At the date of final censoring of patient outcome data, there had been no missing data in terms of follow‐up and patients who were still alive were censored.

The clinicopathological characteristics, the distribution of patients within each clinicopathological parameter, and their association with survival in the discovery cohort are outlined in supplementary material, Table S1. The mismatch repair (MMR) protein status for all tumours was assessed by immunohistochemistry using antibodies to MLH1 and MSH2 [10]. At the time of the censoring, there had been 309 (47.5%) deaths. The median survival was 103 months (95% CI = 86–120 months), the mean survival was 115 months (95% CI = 108–123 months), and the median follow‐up time, calculated by the ‘reverse Kaplan–Meier’ method, was 88 months (95% CI = 79–97 months).

A tissue microarray was constructed from the primary colorectal tumours which also included 50 normal colon mucosal samples (acquired from at least 10 cm in distance from each tumour). The tissue microarray included two representative 1 mm cores for each sample [11].

Ethical approval for the use of colorectal tissue samples was given by the Grampian Biorepository scientific access group committee (tissue request no. TR000169).

### Validation patient cohort

A publicly available dataset, accession number GSE39582, was used as the validation cohort [12]. It was accessed through the NCBI Gene Expression Omnibus (http://www.ncbi.nlm.nih.gov/geo/). Data for the following clinicopathological characteristics are outlined in supplementary material, Table S2. For better comparison with the discovery cohort, stage IV cases (*n* = 60) and cases with no survival information (*n* = 4) were excluded (i.e. 502 patients included). There were 151 deaths (30.1%), the median survival was 183 months (95% CI = 92–273 months), the mean survival was 130 months (95% CI = 121–140 months), and the median follow‐up time, calculated by the ‘reverse Kaplan–Meier’ method, was 73 months (95% CI = 69–77 months).

The expression values of genes (Affymetrix U133 Plus 2.0 chips) corresponding to the biomarker signature identified in the discovery cohort were obtained using Geo2R (https://www.ncbi.nlm.nih.gov/geo/geo2r/). Each probe ID was mapped to the Entrez gene ID with the corresponding platform files. Only probes that are unique to the target gene were used and, if available, exemplar sequence probes were used. If more than one probe was used for the same gene, the expression values showing the most significant variations were used. To allow comparison with immunostaining scores, gene expression values were assigned to one of the four categories based on three quartiles (25th percentile, 50th percentile, and 75th percentile).

The validation cohort was used to evaluate the prognostic performance of biomarkers signature after the discovery process was completed. Therefore, only values for genes in the signature were obtained and evaluated in the validation cohort.

### Biomarkers

The study used a series of biomarkers (*n* = 66) which had been evaluated in the discovery patient cohort. The names of proteins, associated pathways, immunohistochemistry platforms, methods of scoring, and corresponding antibodies are all detailed in supplementary material, Table S3. The biomarker list includes targets which belong to key pathways implicated in tumour progression such as immune response, metabolism, cell cycle, proliferation, cytoskeleton, and cell migration.

The majority of biomarkers were evaluated using antibodies developed in our laboratory (*n* = 37), while the remainder were assessed using commercially available antibodies (*n* = 29). All biomarkers were assessed by immunohistochemistry, the majority of which were stained using a DAKO autostainer (Dako EnVision™ system; Dako, Ely, UK), with the remaining stained using either a Ventana BenchMark XT (Ventana Medical Systems, Oro Valley, AZ, USA) or a Leica Bond RX (Leica Biosystems, Wetzlar, Germany). Further details of materials and methods are provided in Supplementary materials and methods and supplementary material, Tables S4–S6.

The immunostaining results were assessed by either a semi‐quantitative scoring system [10, 11, 13, 14, 15] or a quantitative automated image analysis [16, 17, 18]. The cores were recorded as missing if they were damaged/folded during the staining process, or if they did not contain tumour cells. Details of the immunohistochemistry assessment and scoring systems are provided in Supplementary materials and methods.

### Biomarker discovery methodology

#### Screening and grouping

**Figure 1:**
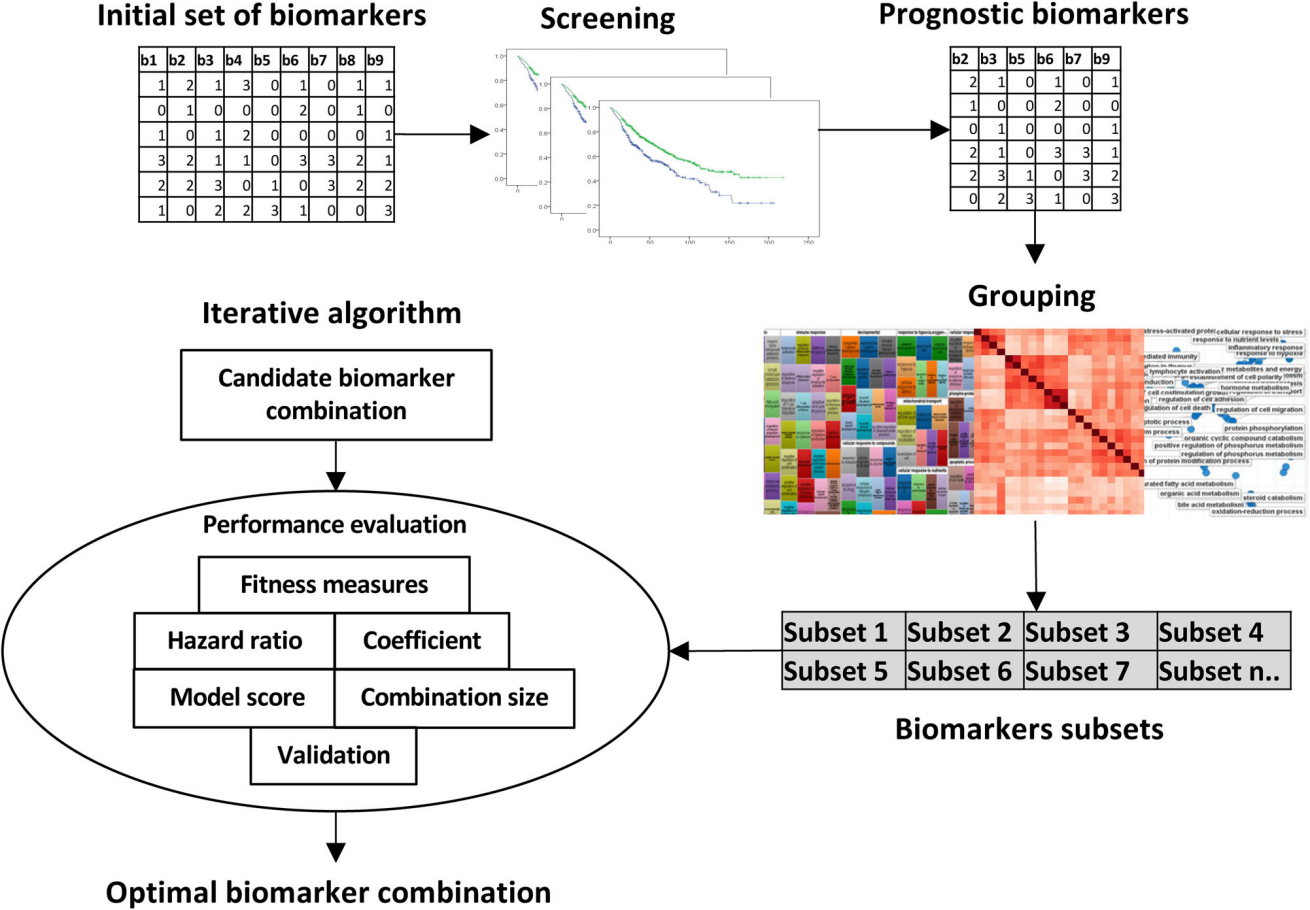
Schematic flowchart of the discovery method used for identifying an optimal prognostic signature. All biomarkers were first screened to determine their individual prognostic power. Then, only prognostic biomarkers were selected and were assembled into subsets based on a range of interrelationships: biological, clinical, and statistical (correlation). The number (*n*) of subsets is dependent on the size of data and number of markers and associated pathways (there were 12 subsets in this study). Each subset was entered into a combinatorial iterative algorithm to identify the best biomarker combination based on a range of fitness measures: HR, combination coefficient, number of biomarkers, multivariate model score, and performance in a validation cohort.

Before applying the iterative algorithm, screening and grouping analysis was performed on the initial series of biomarkers (Figure 1). The screening process involved assessing the survival association of each individual biomarker and removing those with no prognostic power. Then, the remaining biomarkers were assembled into subsets based on a range of interrelationships: biological (homology/family, function, GO term, and pathway analysis); clinical (i.e. whether higher expression of biomarkers is associated with better or worse prognosis); and expression patterns (correlated biomarkers). For the biological groupings, biomarkers can be relevant in different pathways and therefore the same biomarker can be placed in different groups. Each subset was then entered sequentially into the programme to evaluate the best biomarker combination. This deliberate screening and subgrouping of biomarkers improves the probability of identifying robust combinations by providing a biological and clinical framework for interpreting potential discoveries. Limiting the analysis to smaller subsets of biomarkers also reduces the effect of combinatorial explosion.

#### Combinatorial algorithm

The study developed an iterative algorithm to execute the combinatorial analysis on biomarkers (*n*) and identify a combination (C*) with a number of biomarkers (*r*) and a maximum combined fitness (*f*). The fitness in this study represents the prognostic performance of a candidate combination and is mainly evaluated using multivariate Cox regression models which include established prognostic parameters: age, extramural venous invasion (EMVI), lymph node stage and T stage, or age and pathological classification parameter which is based on lymph node stage, T stage, EMVI, and tumour differentiation. The concordance index score was used as a fitness score to evaluate the overall performance of multivariate models (i.e. models which include candidate combination and clinical prognostic parameters), whereas the estimated coefficient, *P* value, and hazard ratio (HR) (default) were each used as a fitness score to determine the univariate and multivariate performance of candidate combinations.

To facilitate a simple iterative evaluation of *f*, a composite variable was computed through the multiple linear equation W1*X1+W2*X2…+Wr*Xr: the total sum of coefficients (*W*) multiplied by the immunostaining scores (*X*) for each biomarker in a combination (coefficients are calculated using Cox regression in a multivariate model which includes *r* biomarkers in a candidate combination).

The combinatorial algorithm first generates biomarker combinations using itertools Python module. For a number *r* ranging from 1 to *n*, and increasing by step size *s*, the code computes every possible combination of *r* biomarker from the *n* list of targets included in our study. We then applied a fitness function to determine the best combination and the best fitness. The number of combinations of *r* biomarkers is calculated by itertools function (itertools.combinations) which uses the following formula: $n!/\left[r!*\left(n-r\right)!\right]:r=\left[1,1+s,\ldots ,n\right],s\in \left\{1-n-1\right\}$. The same process is repeated until convergence. If *s* = 1, then combinations will always have size *n* − 1 which means one biomarker will be eliminated each iteration until convergence. If the size of the initial population is small, then larger step (*s*) is preferred because it minimises the chances of getting trapped at local optimal. The algorithm is outlined in Supplementary materials and methods, and the details of the implementation code are available at GitHub repository (https://github.com/aibiologics/cancer_markers). Future modifications and versions of the algorithm will be available at the same GitHub address.

#### Data processing and statistical analysis

The immunostaining scores of biomarkers screened in the discovery cohort were tabulated in an Excel spreadsheet. SPSS (version 25; IBM, Portsmouth, UK) and Python 3.8.6 were used for data analysis and PyCharm Community Edition 2020.3.5 x64 for code editing and testing. A probability value of ≤0.05 was regarded as significant. Further details are outlined in Supplementary materials and methods.

## Results

### Screening and grouping of biomarkers

Survival associations of biomarkers and optimal cut‐off points are shown in supplementary material, Table S7. In the initial biomarker population (*n* = 66), 28 showed significant univariate association with survival and were retained for combinatorial analysis while 38 biomarkers were excluded from further analysis. The majority of biomarkers (*n* = 19) had high expression that was associated with better survival, while the remaining biomarkers had high expression that was associated with poor survival. The best performance of biomarkers as an individual prognostic parameter (i.e. the ability to distinguish between prognostic groups, chi‐square value, *P* value, HR, and finally the prognostic independence in relation to clinical parameters) was observed in relation to CD3, FOXP3, ICOS, CYP8B1, CYP39A1, LIMK2, PTEN, STAT1, and UCP1. The expression profiles of biomarkers in primary tumour, normal colonic mucosa, and in different tumour stages are highlighted in Supplementary materials and methods, Supplementary results, and supplementary material, Figure S1 and Table S7.

Before performing any combinatorial analysis on the remaining biomarkers, they were assembled into subsets. Pathway and GO term analysis showed that biomarkers could be incorporated into six biomarker subsets: metabolism, immune response, response to environment, development, cell death and proliferation, and amalgamation group (adhesion and migration, signalling, phosphorylation, and cytoskeleton) (supplementary material, Figure S2). SPATA2L was excluded due to lack of association with any of the above pathways. Correlation analysis identified four main groups of correlated biomarkers (supplementary material, Figure S3). Based on their survival association (i.e. association of higher expression or lower expression with better or worse survival), biomarkers were divided into two subsets: the majority of biomarkers (*n* = 19) with higher expression that is associated with better prognosis and a smaller group (*n* = 8) mainly composed of cytochromes P450, with higher expression that is associated with worse prognosis (supplementary material, Table S8). Therefore, the iterative algorithm was performed on each of the six biological/pathways subsets, the four main correlations subsets, and the two subsets based on the direction of survival associations. Analysis was also performed on proteins from associated subsets such as response to microenvironment and immune response.

### Optimal biomarker signature

**Figure 2:**
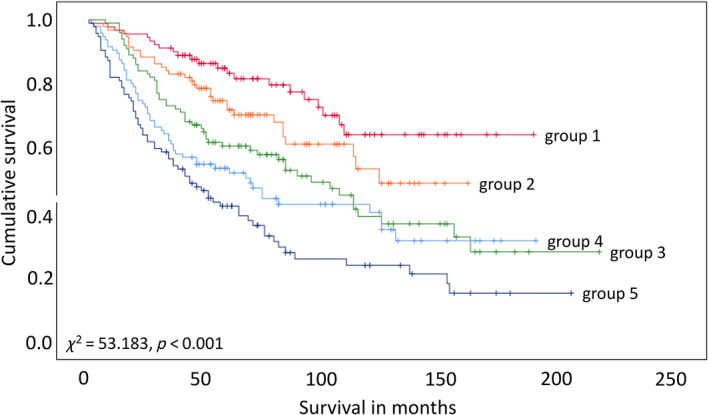
Survival plots of the biomarker signature represented using a linear combination. A signature variable was calculated using the following linear equation: −0.148*FOXP3−0.158*ICOS−0.091*LIMK2+0.137*p‐cofilin−0.038*STAT1−0.164*UCP1. Five groups were computed using four cut‐offs points at equal percentiles. Group 1 was used as the reference group for comparison with groups 2–5. Group 1 versus group 2, *p* = 0.084, *χ* ^2^ = 2.978, HR = 1.595 (95% CI = 0.930–2.737); group 1 versus group 3, *p* < 0.001, *χ* ^2^ = 13.442, HR = 2.422 (95% CI = 1.475–3.977); group 1 versus group 4, *p* < 0.001, *χ* ^2^ = 21.646, HR = 3.061 (95% CI = 1.866–5.021); group 1 versus group 5, *p* < 0.001, *χ* ^2^ = 43.098, HR = 4.383 (95% CI = 2.708–7.095).

Running the iterative algorithm employing a range of parameters and manually evaluating the solutions based on predetermined criteria (supporting information material and methods S2), an optimal biomarker combination was identified from the biological subset: ‘response to environment’ (supplementary material, Figure S2). The biomarker signature consists of six biomarkers: FOXP3, ICOS, LIMK2, p‐cofilin, STAT1, and UCP1. The signature is represented by a composite variable computed with the following equation: −0.148*FOXP3−0.158*ICOS−0.091*LIMK2+0.137*p‐cofilin−0.038*STAT1−0.164*UCP1 (each biomarker is replaced by its corresponding immunostaining score). The composite variable (computed with the above linear formula) was divided into five different patient groups using four cut‐off points of equal percentiles. The signature composite variable was significantly associated with survival (*χ* ^2^ = 53.183, *p* < 0.001; Figure 2). There were significant differences in prognosis between group 1 and group 3 (HR = 2.422, 95% CI = 1.475–3.977, *χ* ^2^ = 13.442, *p* < 0.001), group 1 versus group 4 (HR = 3.061, 95% CI = 1.866–5.021, *χ* ^2^ = 21.646, *p* < 0.001), and group 1 versus group 5 (HR = 4.383, 95% CI = 2.708–7.095, *χ* ^2^ = 43.098, *p* < 0.001).

**Figure 3:**
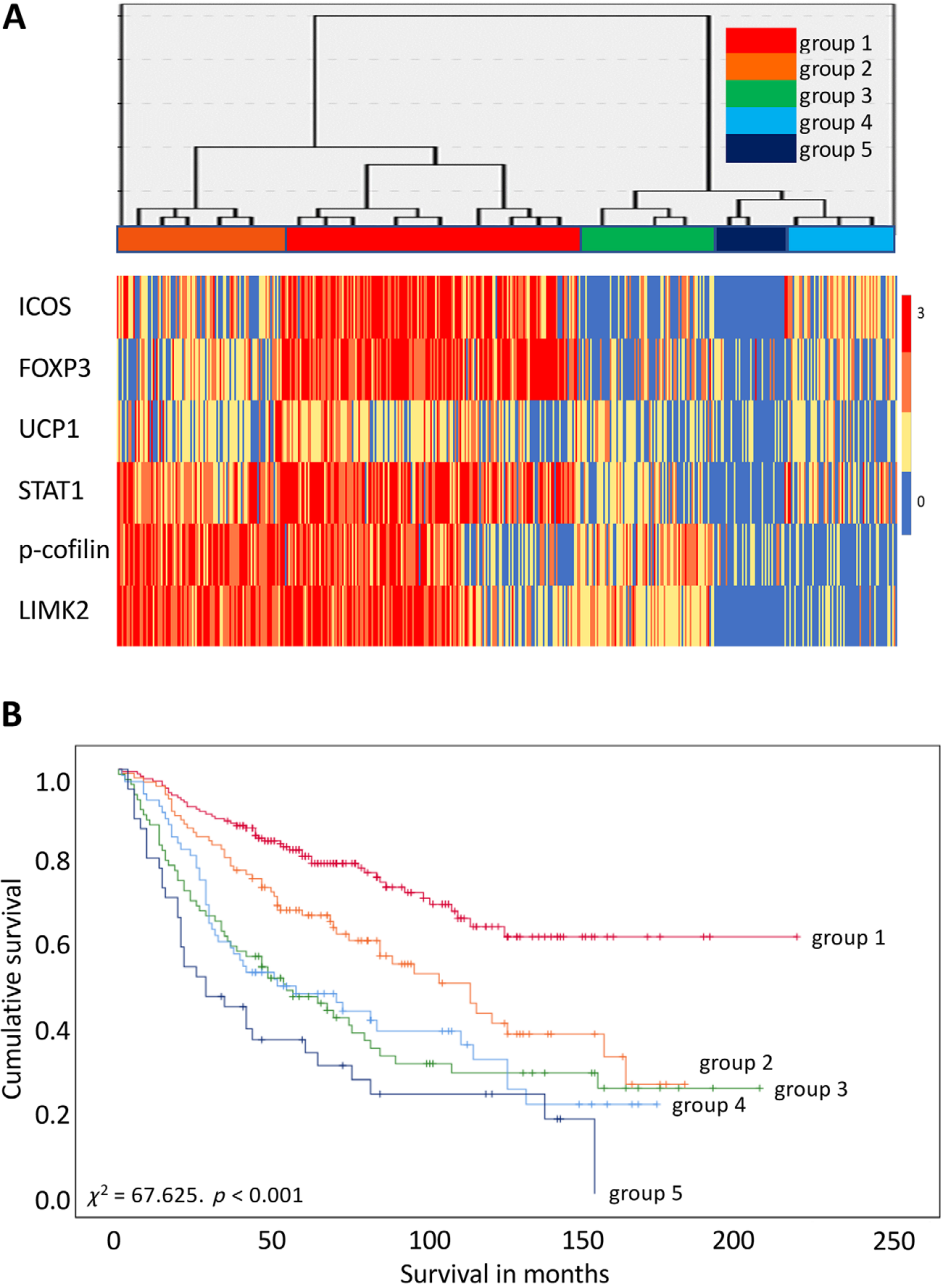
Hierarchical cluster analysis and survival plots of the biomarker signature. (A) Graphical representation of the expression level of biomarkers is shown in the left‐hand panels. The right‐hand panels show the results of the hierarchical cluster analysis presented as a dendrogram with five groups. Biomarkers are represented in columns and individual cases in rows. (B) Survival plots of prognostic groups identified through the biomarker signature. Group 1 was used as the reference group for comparison with the other groups (groups 2–5). Group 1 versus group 2, *p* = 0.001, *χ* ^2^ = 10.693, HR = 1.911 (95% CI = 1.289–2.835); group 1 versus group 3, *p* < 0.001, *χ* ^2^ = 38.382, HR = 3.107 (95% CI = 2.112–4.571); group 1 versus group 4, *p* < 0.001, *χ* ^2^ = 32.243, HR = 3.007 (95% CI = 2.005–4.510); group 1 versus group 5, *p* < 0.001, *χ* ^2^ = 54.454, HR = 4.678 (95% CI = 3.005–7.284).

Hierarchical cluster analysis was also performed to evaluate the expression profile of biomarkers in the above signature. Five prognostic cluster groups with significant differences in survival were identified based on their expression patterns and survival associations (*χ* ^2^ = 67.625, *p* < 0.001; Figure 3). Tumours displaying stronger or higher expression of these biomarkers were associated with better prognosis relative to the those with weaker expression. The median survival of patients was undefined (i.e. if the cumulative survival is more than 50% of patients at the last time point, the median survival cannot be calculated) for group 1 (*n* = 181), 103 months (95% CI = 73–133 months) for group 2 (*n* = 101), 53 months (95% CI = 30–76 months) for group 3 (*n* = 84), 51 months (95% CI = 18–84 months) for group 4 (*n* = 69), and 28 months (95% CI = 13–43 months) for group 5 (*n* = 43). The HR of the patients in cluster group 5 (the worst group in terms of survival) was 4.678 (95% CI = 3.005–7.284) relative to group 1 which has the best prognosis (Figure 3).

Multivariate analysis using four models showed that this biomarker signature was independently prognostic in relation to clinical and pathological parameters (Tables 1 and 2). In the first model, the biomarker signature was significantly associated with survival independent of age, T stage, lymph node stage, and EMVI (linear variable; Wald = 32.898, *p* < 0.001 and cluster variable; Wald = 34.014, *p* < 0.001). Further analysis also showed the signature was significantly independent of age, EMVI, and UICC stage in the second multivariate model (linear variable; Wald = 29.438, *p* < 0.001 and cluster variable; Wald = 28.081, *p* < 0.001), and independent of age and the pathological risk parameter in the third model (linear variable; Wald = 35.607, *p* < 0.001 and cluster variable; Wald = 36.620, *p* < 0.001). Finally, in the fourth model, a multivariate analysis was performed using only parameters that would be available at pre‐tumour resection (i.e. biopsy stage where no pathological information is available about tumour stage, lymph node metastasis, or EMVI). In this model, the signature was highly significant and was the best prognostic indicator compared to age, anatomical site of tumour, and tumour differentiation (linear variable; Wald = 51.359, *p* < 0.001 and cluster variable; Wald = 65.708, *p* < 0.001).

**Table 1:** Multivariate analysis of biomarker signature groups obtained using a linear equation

	Multivariate: age, T stage, N stage, and EMVI			Multivariate: age, UICC stage, and EMVI			Multivariate: age and pathological risk classification*			Multivariate (pre‐resection): age, site, and differentiation		
	First group as reference			First group as reference			First group as reference			First group as reference		
Biomarker signature groups	Wald	*P* value	HR (95% CI)	Wald	*P* value	HR (95% CI)	Wald	*P* value	HR (95% CI)	Wald	*P* value	HR (95% CI)
Group 1 (ref)	32.898	**<0.001**	Overall	29.438	**<0.001**	Overall	35.607	**<0.001**	Overall	51.359	**<0.001**	Overall
Group 2	0.720	0.396	1.265 (0.735–2.177)	0.852	0.356	1.292 (0.750–2.223)	1.621	0.203	1.420 (0.828–2.437)	2.120	0.145	1.495 (0.870–2.568)
Group 3	1.338	0.247	1.357 (0.809–2.276)	3.332	0.068	1.607 (0.966–2.675)	5.463	**0.019**	1.817 (1.101–2.999)	10.733	**0.001**	2.304 (1.398–3.796)
Group 4	6.111	**0.013**	1.908 (1.143–3.184)	7.689	**0.006**	2.066 (1.237–3.451)	11.165	**0.001**	2.342 (1.422–3.858)	16.995	**<0.001**	2.870 (1.739–4.738)
Group 5	19.674	**<0.001**	3.101 (1.881–5.114)	19.599	**<0.001**	3.087 (1.874–5.084)	25.570	**<0.001**	3.513 (2.158–5.716)	37.399	**<0.001**	4.507 (2.782–7.303)

Age: <70 versus ≥70, T stage: T1 versus T2 versus T3 versus T4, N stage: N0 versus N1 versus N2, UICC stage: I versus II versus III. Significant values are highlighted in bold.

* Pathological risk classification: low risk (pT1–pT3 and [G1 or G2] and V0 and pN0) versus high risk ([pN0 and pT4 or G3 or V1] or pN1 or pN2), site: proximal versus distal versus rectum and tumour differentiation: G1 or G2 versus G3.

**Table 2:** Multivariate analysis of biomarker signature groups obtained using a clustering analysis

	Multivariate: age, T stage, N stage, and EMVI			Multivariate: age, UICC stage, and EMVI			Multivariate: age and pathological risk classification*			Multivariate (pre‐resection): age, site, and differentiation		
	First group as reference			First group as reference			First group as reference			First group as reference		
Biomarker signature groups	Wald	*P* value	HR (95% CI)	Wald	*P* value	HR (95% CI)	Wald	*P* value	HR (95% CI)	Wald	*P* value	HR (95% CI)
Group 1 (ref)	34.014	**<0.001**	Overall	28.801	**<0.001**	Overall	36.620	**<0.001**	Overall	65.708	**<0.001**	Overall
Group 2	2.544	0.111	1.395 (0.927–2.100)	5.053	**0.025**	1.585 (1.061–2.368)	7.447	**0.006**	1.733 (1.168–2.573)	8.672	**0.003**	1.811 (1.220–2.688)
Group 3	12.019	**0.001**	2.086 (1.377–3.161)	13.758	**<0.001**	2.166 (1.440–3.258)	17.758	**<0.001**	2.337 (1.575–3.469)	31.566	**<0.001**	3.049 (2.067–4.499)
Group 4	4.852	**0.028**	1.636 (1.056–2.535)	8.166	**0.004**	1.869 (1.217–2.869)	14.789	**<0.001**	2.248 (1.488–3.397)	28.704	**<0.001**	3.046 (2.027–4.579)
Group 5	31.528	**<0.001**	3.848 (2.404–6.158)	26.597	**<0.001**	3.374 (2.125–5.357)	35.031	**<0.001**	3.903 (2.486–6.128)	52.595	**<0.001**	5.236 (3.347–8.191)

Age: <70 versus ≥70, T stage: T1 versus T2 versus T3 versus T4, N stage: N0 versus N1 versus N2, UICC stage: I versus II versus III. Significant values are highlighted in bold.

* Pathological risk classification: low risk (pT1–pT3 and [G1 or G2] and V0 and pN0) versus high risk ([pN0 and pT4 or G3 or V1] or pN1 or pN2), site: proximal versus distal versus rectum and tumour differentiation: G1 or G2 versus G3.

The composite biomarker signature developed using the discovery cohort was next applied to the external validation cohort to stratify patients into prognostic groups. The signature variable was significantly associated with survival (*χ* ^2^ = 14.217, *p* = 0.007; supplementary material, Figure S4) and was prognostically independent in a multivariate model including age, tumour stage, and lymph node stage (Wald = 9.849, *p* = 0.043) and a model including age and TNM stage (Wald = 13.077, *p* = 0.011).

The relationships between the biomarker signature and clinicopathological parameters were also investigated (supplementary material, Figures S5, Figure S6, and Table S9). A proportion of patients, classified based on clinically established pathological parameters, would have different prognosis using the biomarker signature (supplementary material, Figure S5 and Table S9). Groups 1 and 2 identified through the biomarker signature as good prognostic groups include 35% of patients who are classified as high risk based on the established pathological evaluation. Similarly, the prognostic evaluation of a proportion of patients using tumour stage, lymph node involvement, or UICC stage would be significantly different if the biomarker signature is considered (supplementary material, Figure S6, Table S9, and Table S10).

## Discussion

Colorectal cancer is a common tumour with an incidence rate that is rising and with a mortality rate that is still relatively high [2]. There have been considerable improvements to our understanding of the molecular pathways underpinning the development and progression of colorectal cancer [5, 19]. However, only a small number of biomarkers (e.g. KRAS mutations, BRAF mutations, and microsatellite instability status) have been translated into routine clinical practice. Therefore, there is a clear requirement to identify molecular signatures that are clinically useful in improving the accuracy of prognosis and potentially applied as tools for screening, early diagnosis, and therapy of colorectal cancer [6, 7].

In this study, a set of novel biomarkers was assessed by immunohistochemistry using well‐characterised cohort of colorectal cancers. The results of immunostaining provided valuable insight into the expression profiles of proteins, many of which are studied for the first time in colorectal cancer. Significant patterns of increased and decreased expression were observed for a number of biomarkers in primary colorectal cancer compared to normal colonic mucosa. A number of proteins showed significant univariate association with overall survival: CDX2, cytochrome P450, LMK2, PTEN, STAT1, T‐cell markers, and UCP1. This is consistent with previous studies implicating these biomarkers in tumour progression and prognosis [10, 15, 16, 18, 20, 21, 22].

Due to the large number of molecules that can be classified as a biomarker, an extremely large number of combinations would have to be evaluated to identify the relevant combination. This study developed an effective approach for performing combinatorial analysis and the selection of optimal prognostic combinations. The method consists of a screening and grouping analysis, which is designed to minimise the ‘combinatorial explosion’ and reduce the false discovery rate. The screening restricts the combinatorial analysis to biomarkers with significant individual performance [23]. The grouping step further reduces the number of potential combinations by allocating targets into smaller subsets based on their biological relationships, clinical associations, and expression patterns. This also provides a clinical, biological, and molecular framework for interpreting biomarker combinations within each subset.

The second element of the discovery approach involves inputting each biomarker subset into a combinatorial programme, which computes all combinations using itertools Python module and assesses their prognostic power. Using this method, the optimal solution is found when using the global approach (generating all combinations with the formula $n!/\left[r!*\left(n-r\right)!\right]$, where the number of marker *r* in each combination ranging from 1 to all markers in subset [*n*]). This approach is computationally intensive and may not be possible with larger number of variables. Therefore, this study customised the algorithm to incorporate a gradient approach whereby one biomarker is removed at each stage meaning combinations will always have size *n* − 1. The gradient approach is much quicker, but the solution is not the best, as the algorithm is normally trapped at local optimum. In addition to gradient and global parameters, the programme incorporated a wide range of parameters for missing interpolation, multivariate and univariate analysis, model versus single variable evaluation, and internal validation. This algorithm provides a comprehensive exploration of data and can efficiently generate solutions that can be relevant in a wide range of biomarker studies. However, additional customisation, optimisation, and more automation are needed to improve the efficiency and applicability of the algorithmic programme. Specific consideration is needed to the computation of the composite variable which is currently calculated using either a linear equation or a clustering. Alternative methods can be easily implemented using a range of multidimensionality reduction methods such as factor analysis and structural equation modelling. Another issue is the identification of an optimal number of groups (i.e. optimal dichotomisation of the patients) and cut‐off points. In this study, to avoid bias, the variables were all dichotomised using the same method (i.e. equal binning).

**Figure 4:**
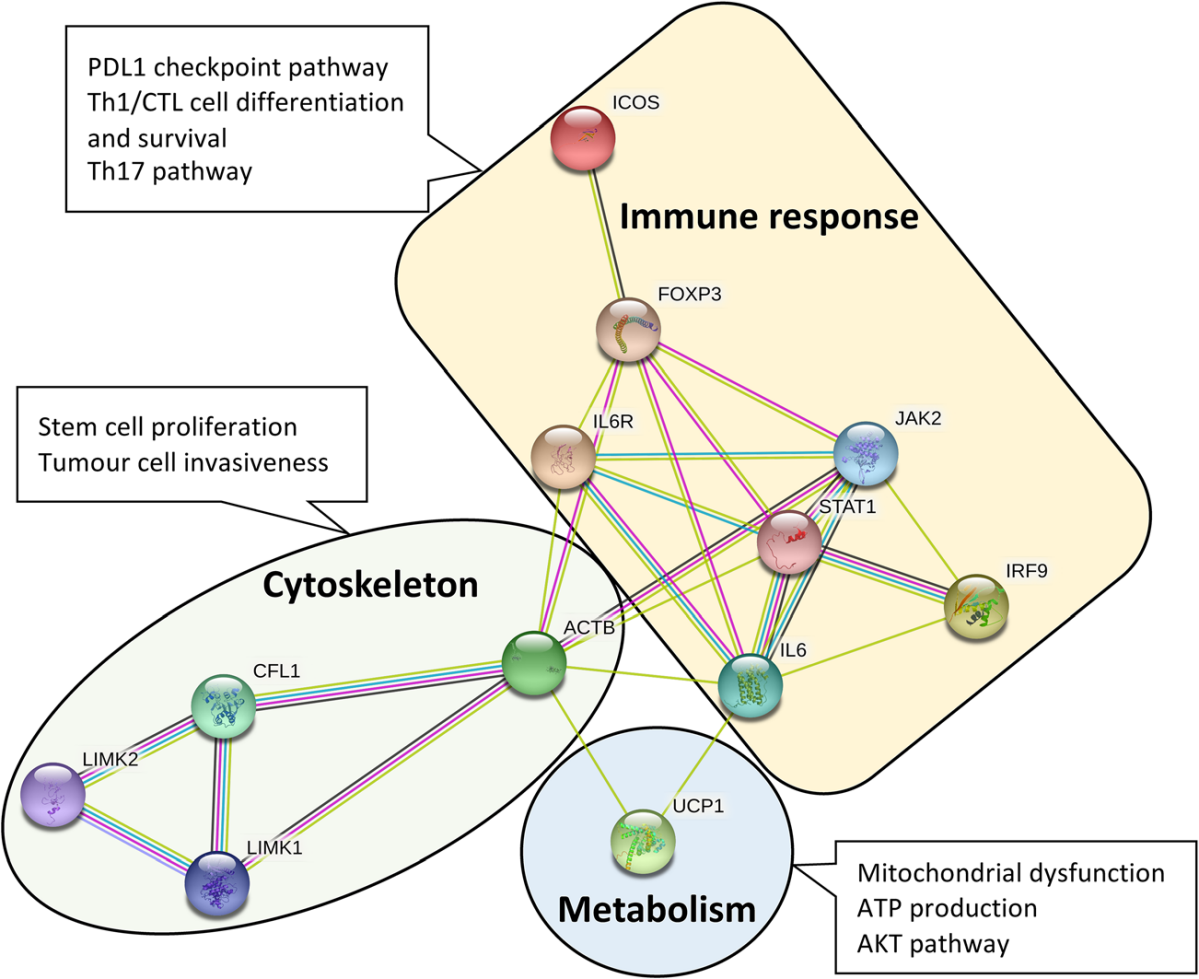
Network node analysis of known and predicted interactions between proteins in biomarker signature. The network was built using STRING (https://string‐db.org/) and KEGG mapper (https://www.genome.jp/kegg/mapper.html). Search is performed using multiple proteins and listing their names (FOXP3, ICOS, LIMK2, CFL1 [symbol of p‐cofilin], STAT1, and UCP1). IL6 was added to the list for its known interaction with all involved pathways. Network type: full network; meaning of network edges: evidence; active interaction sources: text‐mining, experiments, databases, co‐expression, neighbourhood, gene function, and co‐occurrence; minimum required interaction scores: medium confidence. The number of interactions can be adjusted up and down depending on how many interactions are required.

Using the above algorithm, the study identified a prognostic biomarker signature which can divide tumours into different risk subtypes in terms of outcome. The biomarker signature comprised of proteins with complex biological and functional networks mainly associated with immune response, cytoskeletal organisation, and metabolic pathways (Figure 4). The relationships between molecules across these different pathways illustrate the complex nature of microenvironment, especially the immune response and its impact on the outcome of tumour.

The expression of certain proteins can signify whether a specific immune response has either a pro‐tumour or anti‐tumour effects. In our signature, the expression of STAT1 and FOXP3 is the main indicator of anti‐tumour response through sustaining T‐cell population which is tumour suppressive [24, 25]. On the other hand, the expression of ICOS, its association with FOXP3, and its effect on tumour progression are far more complicated. While some studies presented ICOS as a negative predictor of prognosis especially in FOXP3+ T‐regs cells, others demonstrated that higher ICOS expression was associated with better survival in colorectal cancer and lung cancer, when its expression is examined in the context of T‐helper (Th1)/cytotoxic T lymphocytes and in the context of higher CD3 expression rather than T‐regs [16, 26, 27, 28].

The association of this biomarker signature with survival is also affected by rearrangements in the cytoskeleton which is closely linked to the immune response in tumour microenvironment [29]. Higher expression of LIMK2 and p‐cofilin has been associated with anti‐tumour effect through inhibition of stem cell proliferation and tumour cells invasiveness [18]. Furthermore, this signature has a metabolic characteristic which is an established pathway in carcinogenesis [5]. Positive outcome was observed in tumours with higher expression of uncoupling proteins implicated in mitochondrial dysfunction and ATP production [15, 30, 31].

Multivariate analysis confirmed the biomarker signature was prognostically independent of current prognostic methods that are used clinically. There were significant differences in the risk groups of the biomarker signature in terms of median survival and HR. The biomarker signature also showed there could be significant improvement to the accuracy of risk classifications compared to current pathological parameters (tumour differentiation, tumour stage, lymph node stage, and EMVI). Therefore, this signature can potentially be incorporated in the clinical practice as a complimentary factor to the current prognostic methods.

This biomarker signature is even more relevant at the biopsy stage where it demonstrated a strong performance compared to all prognostic parameters that would be available at that stage. This is currently important in rectal cancer due to the increasing use of neoadjuvant therapy followed by either active surveillance follow‐up or salvage surgery [32]. Prognostic molecular tools could be essential for determining initial treatment for cases based on biopsies.

Furthermore, the biomarker signature was prognostically significant in MMR‐proficient tumours, which represents the majority of colorectal cancer. Most of the existing and ongoing immunotherapies (e.g. anti‐CTLA4 and anti‐PD1) are directed towards MMR‐defective tumours [33]. Therefore, the identification of subsets of MMR‐proficient tumours with specific molecular signatures will help guide potential treatment strategies and novel targeted therapies in this group.

The findings of this study might be limited by the inclusion of biomarkers with different scoring methods (quantitative versus semi‐quantitative) and hence the resulting dichotomisation of quantitative scores might not accurately mirror the semi‐quantitative scores of negative, weak, moderate, and strong. Moreover, the algorithm and corresponding codes need further customisation and optimisation before efficiently identifying optimal combinations without manual adjustments of input and parameters based on outputs. The code is publicly available on GitHub to use, optimise, adapt, comment upon, and provide feedback. Future modifications and new versions of the code will be available at the following GitHub address (https://github.com/aibiologics/cancer_markers).

To conclude, this study has developed an effective exploratory method with a range of algorithmic parameters designed to identify optimal combinations of biomarkers based on their prognostic power in terms of subtyping tumours prognostically. Using this method, a novel biomarker signature with strong prognostic power in colorectal cancer was identified. This signature could potentially act as a prognostic parameter which is complimentary to the existing prognostic methods. Furthermore, the findings further highlight the molecular complexity of cancer and its microenvironment and provide a panel of actionable targets that can be manipulated therapeutically to supress tumour progression.

## Author contributions statement

AbA wrote the first draft and edited the manuscript, performed the experimental work (immunohistochemistry and scoring), processed and analysed the data, developed the algorithm, and wrote the corresponding Python codes. WP and MI edited, optimised, and consolidated the Python codes in GitHub. TW and AyA performed and supervised the experimental work in relation to antibody development and validation. SGC, MPH, KM, and MST performed the experimental work (immunohistochemistry and immunostaining scoring) in relation to the immune markers and contributed to editing of the manuscript. GIM initiated, supervised, and led the project; designed the experiments; edited and revised the manuscript; and guided all aspects of this study.

## Supporting information


Supplementary materials and methods
Supplementary results
**Figure S1.** Frequency distribution of the immunostaining scores in primary colorectal cancer and normal colon mucosa for antibodies that have not been previously published
**Figure S2.** Treemap representation of the main pathways associated with 29 biomarker targets included in the combinatorial analysis
**Figure S3.** Correlation matrix of biomarkers included in combinatorial analysis
**Figure S4.** Kaplan–Meier survival analysis of the biomarker signature in the validation cohort
**Figure S5.** Distribution of the pathological risk groups across the prognostic groups of biomarker signature
**Figure S6.** Distribution of UICC stage, T stage, lymph node stage, and MMR status across the prognostic groups of the signature
**Table S1.** Clinicopathological characteristics of patients in the discovery cohort (Grampian cohort), their tumours, and the relationship of each variable with overall survival
**Table S2.** Clinicopathological characteristics of patients in the validation cohort, their tumours, and the relationship of each variable with overall survival
**Table S3.** Biomarkers screened in the discovery cohort of colorectal cancer
**Table S4.** Peptide sequences used as immunogens to generate monoclonal antibodies which have not been previously tested on the Grampian patient cohort
**Table S5.** The numbers of normal and tumour tissue samples in the multi‐tissue microarray
**Table S6.** Dilutions, antigen retrieval conditions, and subcellular localisation of antibodies which have not been previously published
T**able S7.** Comparison of the expression of each protein in normal colonic mucosa and different UICC stages in primary colorectal cancer using antibodies that have not been previously published in the discovery cohort
**Table S8.** Starting list of biomarkers included in the biomarker combinatorial analysis
**Table S9.** Associations of the biomarker signature and clinicopathological variables. Survival analysis of the biomarker signature was stratified by clinicopathological variables
**Table S10.** Comparisons of the clinicopathological characteristics of cases for which current pathological risk classifications differ from the risk groups of the biomarker signature
Remark checklist
