# Supplementary material for: Identification of a prognostic signature in colorectal cancer using combinatorial algorithm‐driven analysis
Source: J Pathol Clin Res. 2022 Jan 18;8(3):245–56. doi: 10.1002/cjp2.258 (PMC8977276; doi:10.1002/cjp2.258)
Supplement: Supplementary file 1 — Supplementary materials and methods Supplementary results Figure S1. Frequency distribution of the immunostaining scores in primary colorectal cancer and normal colon mucosa for antibodies that have not been previously published Figure S2. Treemap representation of the main pathways associated with 29 biomarker targets included in the combinatorial analysis Figure S3. Correlation matrix of biomarkers included in combinatorial analysis Figure S4. Kaplan–Meier survival analysis of the biomarker signature in the validation cohort Figure S5. Distribution of the pathological risk groups across the prognostic groups of biomarker signature Figure S6. Distribution of UICC stage, T stage, lymph node stage, and MMR status across the prognostic groups of the signature Table S1. Clinicopathological characteristics of patients in the discovery cohort (Grampian cohort), their tumours, and the relationship of each variable with overall survival Table S2. Clinicopathological characteristics of patients in the validation cohort, their tumours, and the relationship of each variable with overall survival Table S3. Biomarkers screened in the discovery cohort of colorectal cancer Table S4. Peptide sequences used as immunogens to generate monoclonal antibodies which have not been previously tested on the Grampian patient cohort Table S5. The numbers of normal and tumour tissue samples in the multi‐tissue microarray Table S6. Dilutions, antigen retrieval conditions, and subcellular localisation of antibodies which have not been previously published Table S7. Comparison of the expression of each protein in normal colonic mucosa and different UICC stages in primary colorectal cancer using antibodies that have not been previously published in the discovery cohort Table S8. Starting list of biomarkers included in the biomarker combinatorial analysis Table S9. Associations of the biomarker signature and clinicopathological variables. Survival analysis of the biomarker signature was stratified [file CJP2-8-245-s001.pdf]

Identification of a prognostic signature in colorectal cancer using combinatorial algorithm-driven analysis

A Alnabulsi *et al. J Pathol Clin Res* DOI: 10.1002/cjp2.258

Supplementary material

Reference numbers refer to the list in the main text

Contents

Supplementary materials and methods

Supplementary results

Figure S1. Frequency distribution of the immunostaining scores in primary colorectal cancer and normal colon mucosa for antibodies that have not previously been published

Figure S2. Treemap representation of the main pathways associated with 29 biomarker targets included in the combinatorial analysis

Figure S3. Correlation matrix of biomarkers included in combinatorial analysis.

Figure S4. Kaplan–Meier survival analysis of the biomarker signature in the validation cohort

Figure S5. Distribution of the pathological risk groups across the prognostic groups of biomarker signature

Figure S6. Distribution of UICC stage, T stage, lymph node stage and MMR status across the prognostic groups of the signature

Table S1. Clinicopathological characteristics of patients in the discovery cohort (Grampian cohort), their tumours and the relationship of each variable with overall survival

Table S2. Clinicopathological characteristics of patients in the validation cohort, their tumours and the relationship of each variable with overall survival

Table S3. Biomarkers screened on the discovery cohort of colorectal cancer

Table S4. Peptide sequences used as immunogens to generate monoclonal antibodies which have not been previously tested on the Grampian patient cohort

Table S5. The numbers of normal and tumour tissue samples in the multi-tissue microarray

Table S6. Dilutions, antigen retrieval conditions, subcellular localisation of antibodies which have not previously been published

Table S7. Comparison of the expression of each protein in normal colonic mucosa and different UICC stages in primary colorectal cancer using antibodies that have not previously been published on the discovery cohort

Table S8. Starting list of biomarkers included in the biomarker combinatorial analysis

Table S9. Associations of the biomarker signature and clinicopathological variables. Survival analysis of the biomarker signature was stratified by clinicopathological variables

Table S10. Comparisons of the clinicopathological characteristics of cases for which current pathological risk classifications differ from the risk groups of the biomarker signature

Remark checklist

Supplementary materials and methods

The histopathological processing of colorectal tissue specimens

All the colorectal cancer were received fresh immediately after resection and were dissected, fresh tumour and normal samples taken for the Grampian Biorepository, fixed and sampled for histopathology using a standardised protocol. All the specimens used to construct the tissue microarray were processed and fixed in 10% neutral buffered formalin for at least 48 hours at room temperature. Representative tissue blocks were embedded in wax before tissue slides were cut and stained with haematoxylin and eosin (H&E) for histopathological diagnosis. After examining each H&E stained slide an expert gastro-intestinal pathologist (GIM) selected the appropriate areas of tissue to be sampled for constructing the tumour microarray.

Colorectal cancer tissue microarray

The tissue microarray was constructed using 50 normal colon samples (distance of 10 cm from the tumour) and 650 primary colorectal tumours. The tumour resections were from patients who had undergone elective surgery with curative intent for primary colorectal cancer in the following periods: 1994-1998 (n=99), 1999-2003 (n=198) and 2004-2009 (n=353). After identifying suitable areas to be sampled from representative blocks, two cores (1mm in diameter) were taken from each sample and placed in the tissue microarray block.

Immunohistochemistry procedure

The tissue slides were firstly processed as follow: dewaxed in xylene for a minimum of 10 minutes and rehydrated for two minutes by immersion in decreasing ethanol concentrations. For antibodies that did not need antigen retrieval, the slides were then placed in water ready for immunostaining. For the remaining slides, antigen retrieval was performed by microwaving (800W) for 20 minutes while fully immersed in citrate buffer (pH 6.0) or in EDTA (pH 7.8). The primary antibodies were applied on the slides and incubated for 60 minutes at room temperature. Blocking with peroxidase was applied for 7 minutes after the primary antibody was washed twice using Dako buffer washes. The blocking buffer was washed twice before applying the secondary antibody (peroxidase-polymer labelled goat anti-rabbit/mouse, Envision, Dako) for 30 minutes at room temperature. This was followed by further two washes before adding the diaminobenzidine substrate which was incubated for 7

minutes and washed off with water. The slides were counterstained with haematoxylin for 10 seconds and washed using tap water. The slides were then dehydrated in increasing ethanol concentrations for couple of minutes before being immersed in a xylene and mounted. Antibody diluent was used instead of primary antibodies as a negative control. Appropriate normal tissue known to express the relevant protein was used as a positive control.

Immunohistochemistry assessment

The immunostaining results were assessed by either a semi-quantitative scoring system or a quantitative semi-automated image analysis. The semi-quantitative scoring system was conducted using light microscopy by two independent observers unaware of clinical data. Tumour cores were scored based on the intensity of immunostaining of none (score 0), weak (score 1), moderate (score 2) and strong (score 3).

The automated quantitative image analysis was completed using QuPath, Slidepath or Aperio image software (supplementary material, Table S3). The scores (continuous) were based on H-score which is calculated using the following formula: 3x% strongly stained cells+ 2x% moderately stained cells + 1x% weakly stained. Therefore, to allow for meaningful comparisons with the semi-quantitative categorised data, the quantitative scores were dichotomised into four categories (scores 0, 1, 2 and 3) based on 3 cut-off points (quartiles). Categorical score 0 represented cases with H-score which fall under the first quartile, categorical score 1: between the first and second quartiles, categorical score 2: between the second and third quartiles and score 3: higher than the upper quartile.

The immunostaining was assessed in the tumour epithelial compartment (PTEN and VAV1 were assessed in both the stromal and epithelial tumours), except for the immune markers which were assessed in the tumour stroma (supplementary material, Tables S7 & S8). The scores for each biomarker were recorded in Excel before being combined into one master spreadsheet and transferred to SPSS for analysis.

Development of monoclonal antibodies

Antibodies were produced using hybridomas technology and ELISA screening [15]. Short amino acid sequences (8-12 in length) were used as immunogens and were chosen using a range of opensource bioinformatic software [15]. The sequences of peptides and their location on each protein are specified in supplementary material, Table S3. The specificity and sensitivity of all antibodies, developed in our lab, were evaluated by immunoblotting as

previously described [15]. Antibodies were also validated by immunohistochemistry using comprehensive set of multi-tissue microarray (supplementary material, Table S4). Each antibody was tested without antigen retrieval and with antigen retrieval solution. Antigen retrieval was performed by microwaving the tissue slides for 20 minutes while fully immersed in 10mM citrate buffer (pH 6.0) or in EDTA solution (pH=7.8). The immunostaining profile for each antibody was assessed by an expert consultant pathologist (GIM) and interpreted in the context of existing knowledge of the expression of corresponding protein.

Selection criteria of prognostic biomarker signatures

After running the iteration algorithm, solutions were manually evaluated based on the following criteria: Kaplan–Meier plot, median survival of prognostic groups, chi-square value, p-value, hazard ratio, the number of patients in each prognostic group, and finally evaluate the prognostic significance of the biomarker combination against clinical prognostic parameters), the number of biomarkers in the solution combination (i.e., a smaller number is preferred) and the concordance index using internal and external validation sets. The combinatorial programme by default evaluates the hazard ratio as a fitness score. To evaluate other fitness scores, the function "compute fitness" need to be adjusted. This is done by changing cph1.hazard_ratios_ to cph1. hazards_ (coefficients) or to cph1.summary.

Combinatorial algorithm

```
optimalCombinationFunction (biomarkers, generations, s):
  #  n: number of biomarkers, s ∈ {1- n-1}, biomarkers: input list of biomarkers
  # C: all combinations, c: candidate combination, c*: best combination, f: candidate fitness, f*: best fitness
  compareFunction (biomarkers, s):
     for r in range (n-1, n-s, -1):
        C= itertools.combinations (biomarkers, r)
        for c in C:
           f=functionEvaluate (c)
           if f best:
             f*=f & c*=c
     return c*
  for _ in range (generations):
     c=compareFunction (c*, s)
     if f> f*:
        c*=c
  return c*.
```

Data processing and statistical analysis

The following Python libraries and modules were used: NumPy, Pandas, lifelines, scikit-learn, scikit-survival, SciPy, random, collection, itertools, os and logging.

All quantitative scores were dichotomised into four categories based on three cut-off points (i.e., first, second and third quartiles) to allow meaningful comparison with the remaining semi-quantitative data. There was no other transformation of data or any computations of missing values. The following statistical tests were used: Mann-Whitney U test, Wilcoxon signed rank test, chi-squared test, Spearman correlation, Kaplan–Meier survival analysis (log-rank test), Cox's proportional hazard model (univariate and multivariate) and hierarchical cluster analysis (Ward's method). Cox regression was used to calculate the hazard ratio and estimated coefficient of biomarkers and to compute the concordance index score of fitted models. Train-test split option was implemented for evaluating the performance of prognostic models.

Supplementary Results

Immunostaining profiles of new biomarkers

All antibodies showed immunoreactivity in primary colorectal cancer. Of those tested on normal colonic mucosa, only CK7, SPATA2 and cytoplasmic BCLAF were negative in all normal samples. The majority of biomarkers were localised to the tumour cell cytoplasm except for SPATA2L, SPATA25, BCLAF1, HMGB1, SNTB2 and PRDM16 which showed both nuclear and cytoplasmic immunoreactivities. HER2 was localised to the cell membrane while CDX2 was nuclear. The expression of PTEN, VAV1 and y-H2AX was recorded in both epithelial and stromal compartments, whereas the immunostaining of the remaining biomarkers was limited to tumour cells. There was no evidence of tumour heterogeneity between duplicate cores as shown in whole section immunohistochemistry of a sub-set of tumours [10].

Trends of increased and decreased expression were both observed when comparing primary tumour to normal colonic mucosa and when comparing different tumour stages (supplementary material, Figure S1 and Table S8). The immunostaining was significantly higher in primary colorectal cancer compared with normal colonic mucosa for BAG3, BCLAF1c, DRAM1, DRAM2, ERO1lb, PRDM16c, SLA2, CYP2J2, GCSP, yH2, SNTB2c, HMGB1c, EphB6, SPATA2L, SPATA2, TNFRSF18s and UCP2. Whereas the expression of P53, P21, p-Akt, NOTCH, PRDM16n, PTEN, SNTB2n, HMGB1n, SPTA25n, SPATA25n, STAT1 and VAV1 was significantly less in primary tumour samples. The majority of primary tumours (63%) were CK20+/CK7-/CDX2+, with 35% expressing non-classic phenotype in at least one of the three biomarkers and 2% of tumours showing the opposing immunophenotype of CK20-/CK7+/CDX2- (Figure S1). A proportion of 6% of tumour were HER2 positive. The frequency distributions of each immunostaining category for biomarkers scored using semi-quantitative method are shown in supplementary material, Figure S1.

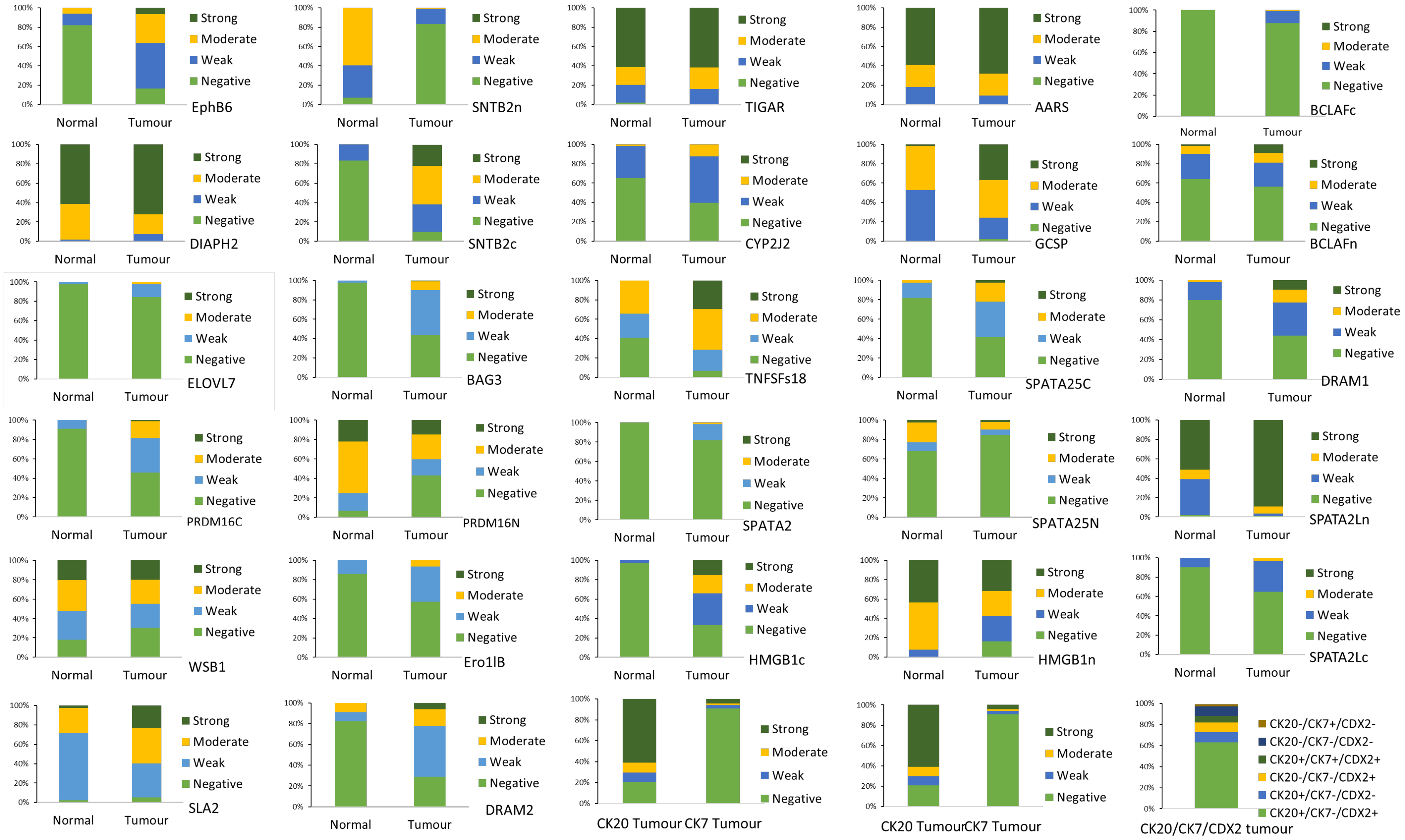

Figure S1. Frequency distribution of the immunostaining scores in primary colorectal cancer and normal colon mucosa for antibodies that have not previously been published. The frequency of negative, weak, moderate and strong immunostaining for biomarkers in normal colonic mucosa and colorectal cancers in the discovery cohort.

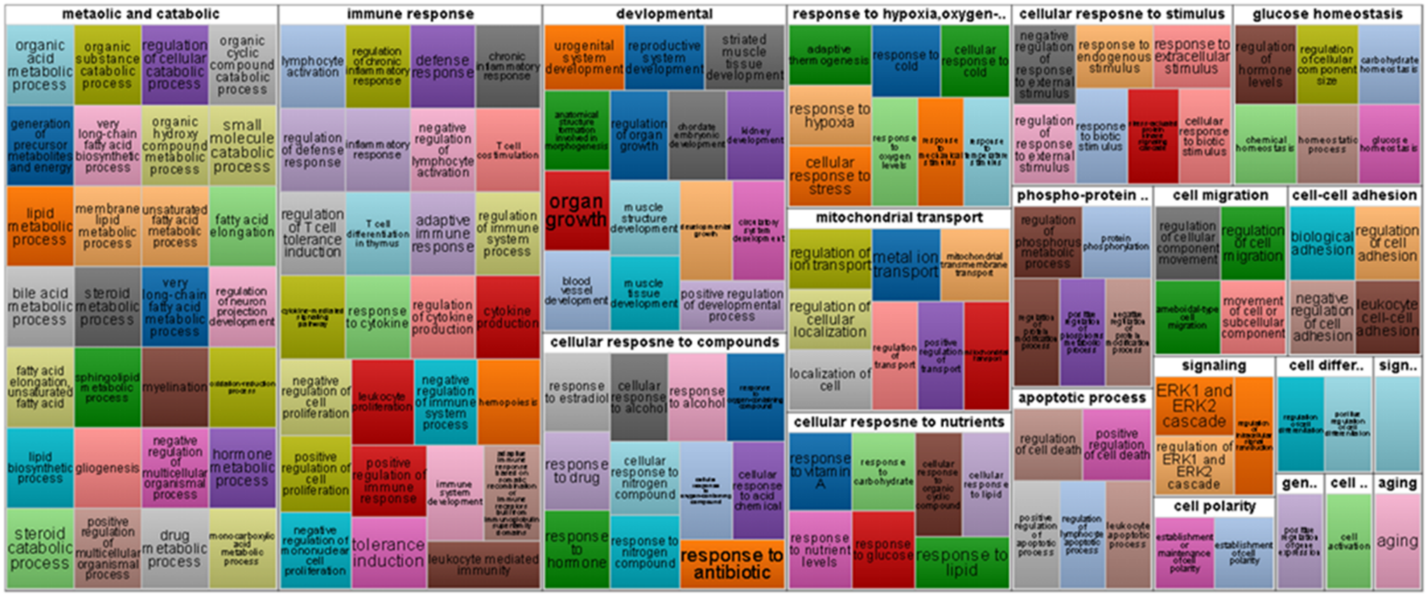

**Response to microenvironment**<br>CD4, STAT1, p-AKT1, UCP1, PTEN, LIMK2, p-cofilin, IDO1, ICOS, FOXP3, and TIAM1	**Metabolism and catabolism**<br>CYP P450 enzymes, ELOVLs, P-AKT, LRAT, PTEN, UCP1, LRAT and CIDEA	**Cell death and proliferation**<br>DRAM1, p-cofilin, p-AKT1, CD3, SPATA2, IDO1, CYP26B1, STAT1, CIDEA, TIAM1 and PTEN
**Development**<br>P-Cofilin, CDX2, CD3,CD4, p-AKT1, IDO1, UCP1, LRAT, UCP2, CYP26B1, CD8, ICOS, LIMK2, FOXP3, TIAM1, PTEN and SPATA2	**Immune response (main T cell)**<br>p-AKT1, CD3, IDO1, CYP26B1, CD4, CD8, ICOS, FOXP3, STAT1, PTEN and p-cofilin	**Phosphorylation, cytoskeleton and adhesion**<br>CD3,IDO1, STAT1, CD4, ICOS, LIMK2, p-cofilin, p-AKT1, TIAM1, PTEN, FOXP3, CD8, CYP26B1

Figure S2. Treemap representation of the main pathways associated with 29 biomarker targets included in the combinatorial analysis. Go term and treemap analyses were performed using online applications: GENERIC GENE ONTOLOGY (GO) TERM FINDER (https://go.princeton.edu/cgi-bin/GOTermFinder) and REVIGO visualisation (http://revigo.irb.hr/). Biomarkers were grouped into six major biological groups; response to environment, metabolism/catabolism, cell death and proliferation, developmental, immune response and cytoskeleton/phosphorylation/adhesion.

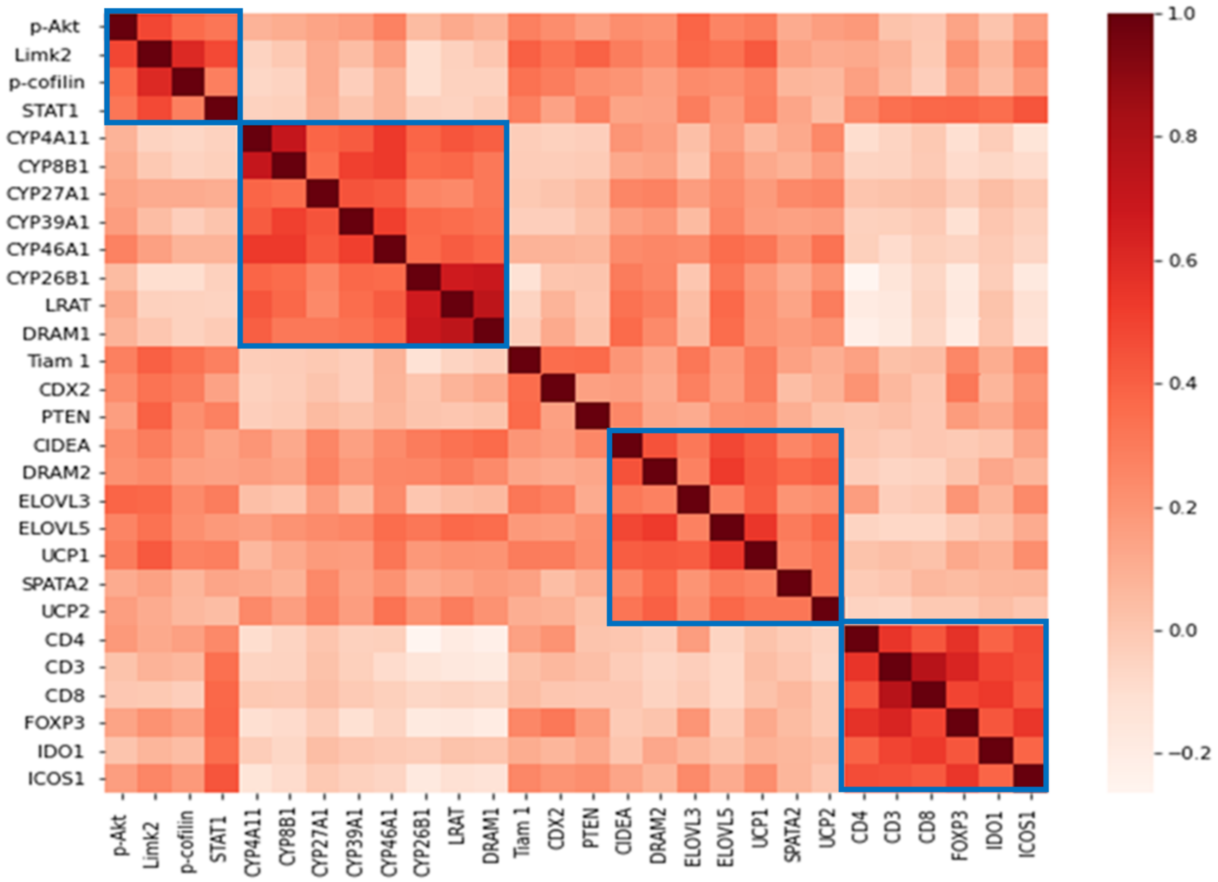

Figure S3. Correlation matrix of biomarkers included in combinatorial analysis. Chi-Square correlation was used to compare relationships between biomarkers. The figure shows four strongly-correlated biomarker subsets; cytochromes P450, immune markers, metabolic and cytoskeleton/signalling.

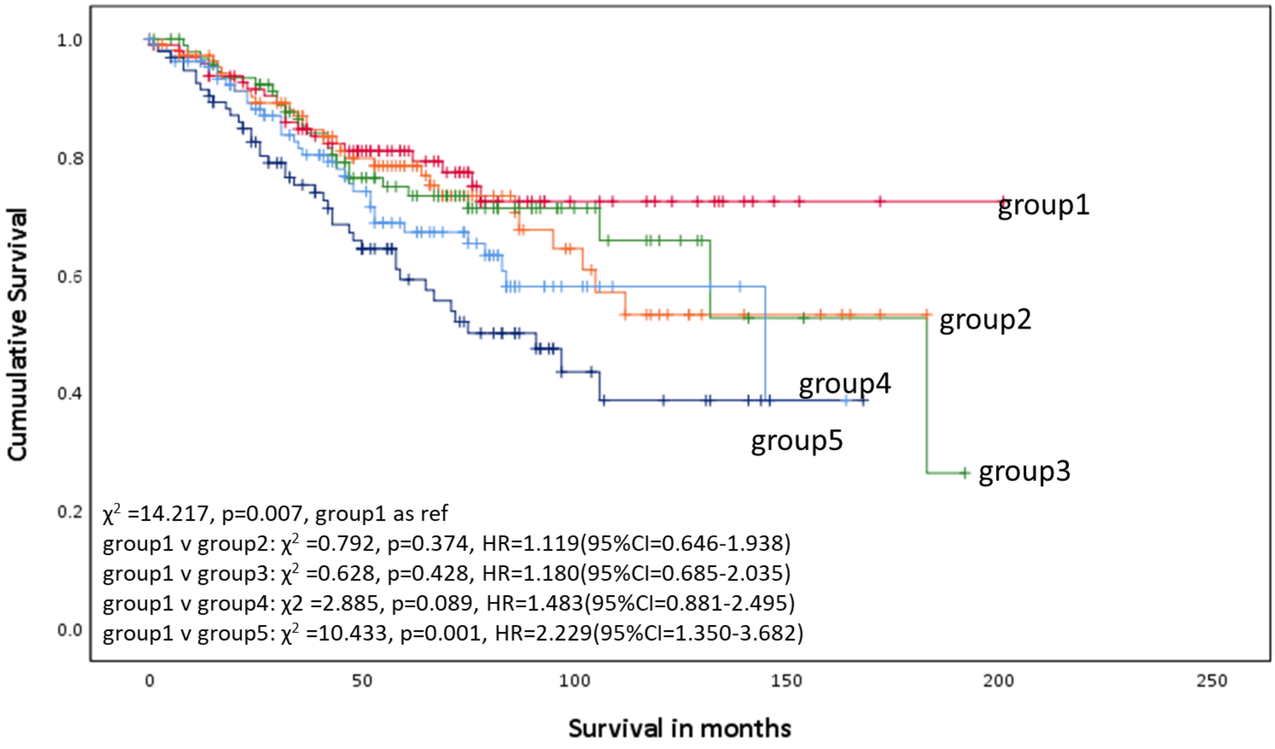

Figure S4. Kaplan–Meier survival analysis of the biomarker signature in the validation cohort.

Survival plots of prognostic groups identified through the biomarker signature in the validation cohort. Group 1 was used as the reference group for comparison with the other groups (groups 2, 3, 4 and 5).

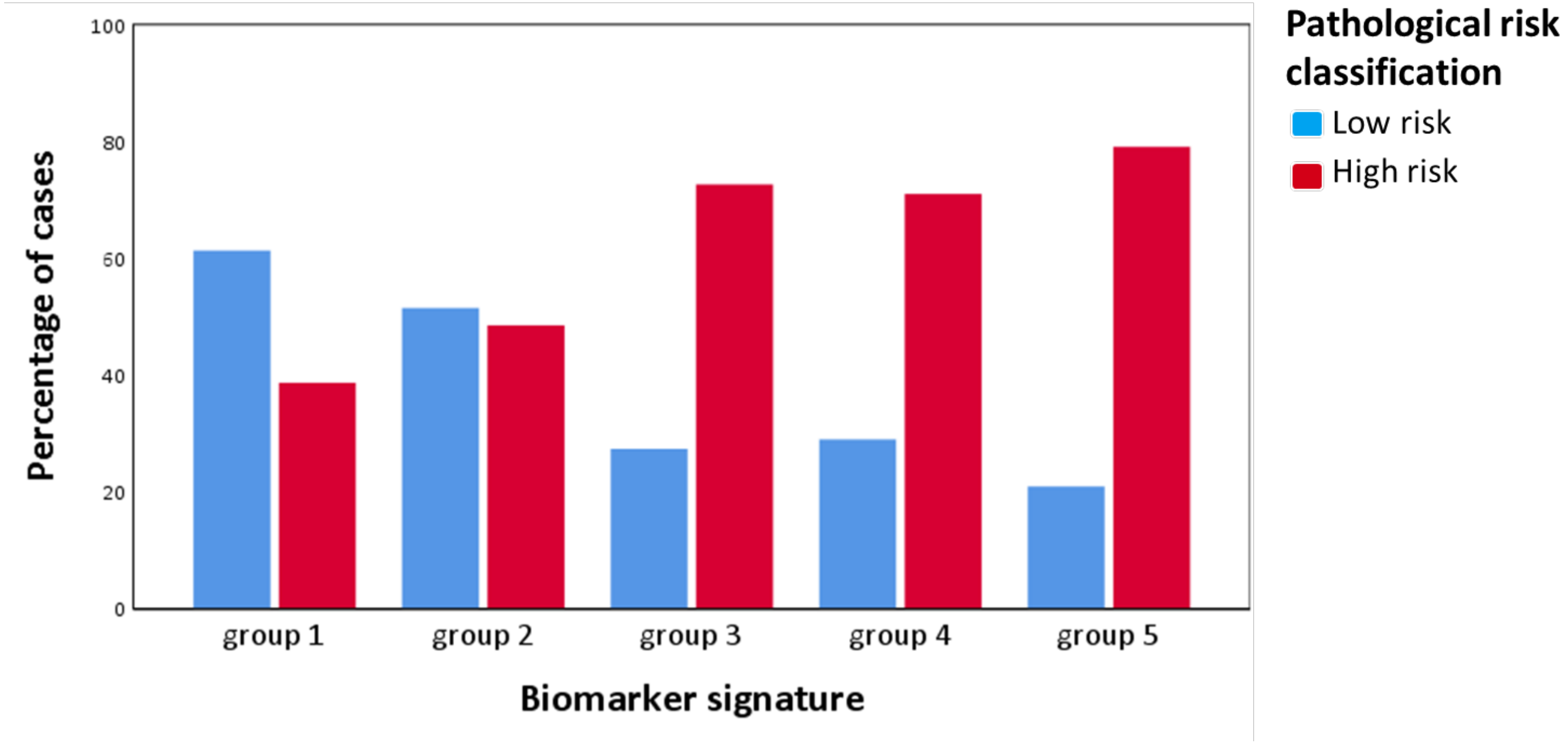

Figure S5. Distribution of the pathological risk groups across the prognostic groups of biomarker signature.

Pathological risk classification: low risk (pT1-pT3 and (G1 or G2) and V0 and pN0) vs high risk ((pN0 and pT4 or G3 or V1) or either pN1 or pN2).

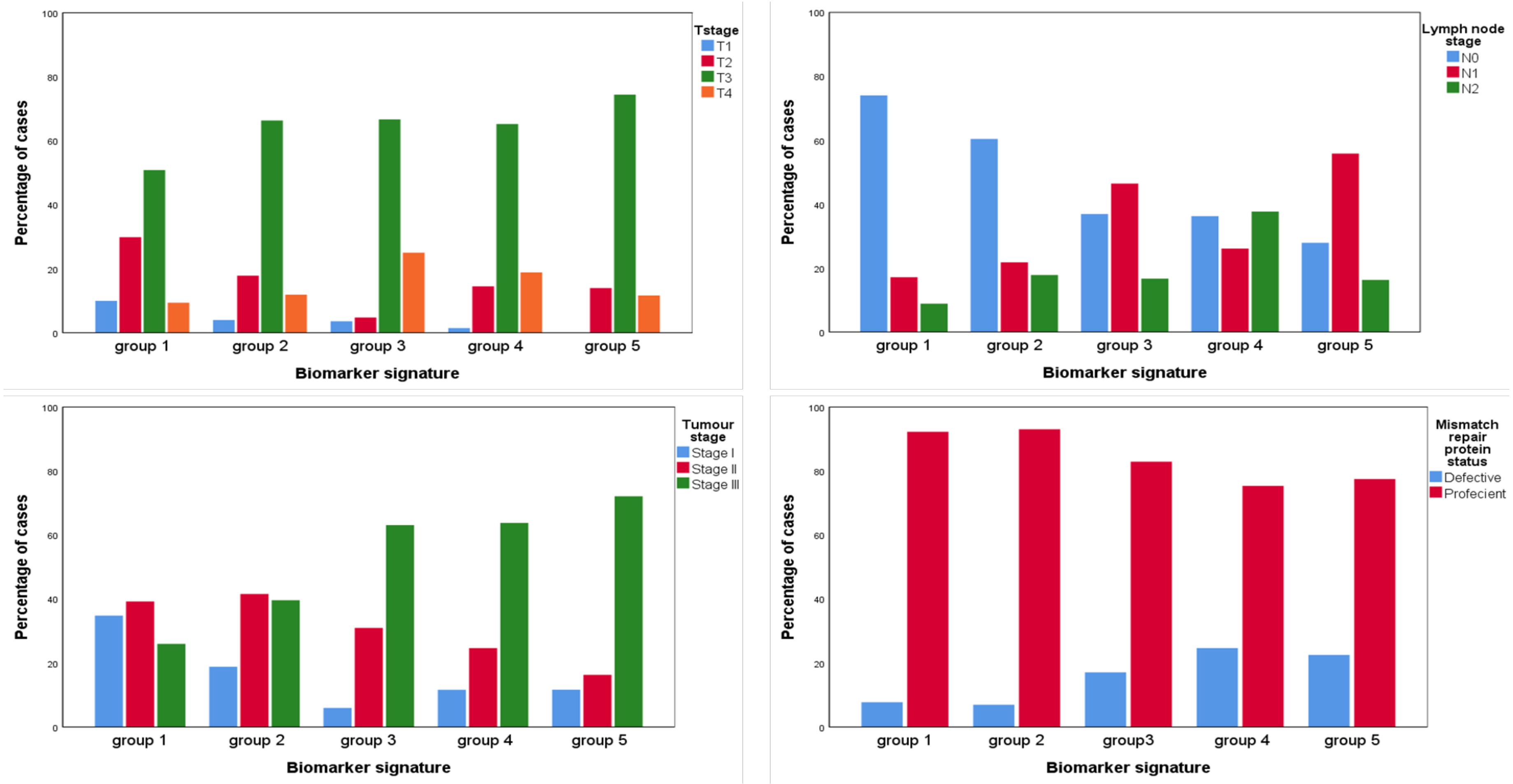

Figure S6. Distribution of UICC stage, T stage, lymph node stage and MMR status across the prognostic groups of the signature.

Table S1. Clinicopathological characteristics of patients in the discovery cohort (Grampian cohort), their tumours and the relationship of each variable with overall survival.

Characteristic	Number	Percent	Relationship with survival
**Sex**			
Male	340	52.3	$\chi2$= 0.027, p=0.870
Female	310	47.7	
**Age**			
<70	305	46.9	$\chi2$=29.213, **p<0.001**
≥70	345	53.1	
**Screen detected**			
Yes	52	8	$\chi2$=16.381, **p<0.001**
No	598	92	
**Tumour site**			
Proximal colon	261	40.2	Proximal v distal, $\chi2$= 8.418, **p=0.004**
Distal colon	245	37.7	Distal v rectal, $\chi2$= 0.906, p=0.341
Rectum	144	22.2	Colon v rectum, $\chi2$=0.098, p=0.754
**Tumour differentiation**			
Well/moderate (G1/G2)	600	92.3	$\chi2$=0.976, p=0.323
Poor (G3)	50	7.7	
**Extra-mural venous invasion**			
Present	140	21.5	$\chi2$=100.946, **p<0.001**
Absent	510	78.5	
**Mismatch repair protein status**			
Defective	96	15.2	$\chi2$=2.848, p=0.091
Proficient	536	84.8	
**T cell marker CD3**			
Low	294	50	$\chi2$=26.445, **p<0.001**
High	293	50	
**pT stage**			
T1	30	4.6	Overall, $\chi2$=78.038, **p<0.001**
T2	114	17.5	T1 v T2, $\chi2$=0.382, p=0.536
T3	411	63.2	T2 v T3, $\chi2$=24.739, **p<0.001**
T4	95	14.6	T3 v T4, $\chi2$=30.159, **p<0.001**
**pN stage**			
N0	364	56	Overall, $\chi2$=142.709, **p<0.001**
N1	177	27.2	N0 v N1, $\chi2$=54.071, **p<0.001**
N2	109	16.8	N1 v N2, $\chi2$=17.636, **p<0.001**
**UICC stage**			
I	120	18.5	Overall, $\chi2$=111.777, **p<0.001**
II	244	37.5	I v II, $\chi2$=5.059, **p=0.025**
III	286	44	II v III, $\chi2$=65.510, **p<0.001**

Significant values are highlighted in bold.

Table S2. Clinicopathological characteristics of patients in the validation cohort, their tumours and the relationship of each variable with overall survival.

Characteristic	Number of	Percentage	Relationship with survival
**Sex**			
Male	278	55.4	$\chi 2$= 3.165, p=0.076
Female	224	44.6	
**Age**			
<70	258	51.4	$\chi 2$=28.889, **p<0.001**
≥70	244	48.6	
**Tumour site**			
Proximal	202	40	$\chi 2$= 0.915, p=0.339
Distal	300	60	
**Microsatellite instability status**			
Defective	70	14	$\chi 2$=0.003, p=0.955
Intact	388	77.3	
NA	44	8.7	
**pT stage**			
T1	14	2.8	Overall, $\chi 2$=14.210, **p=0.007**
T2	39	7.8	T1 v T2, $\chi 2$=0.269, p=0.604
T3	332	66.1	T2 v T3, $\chi 2$=1.064, p=0.306
T4	93	18.5	T3 v T4, $\chi 2$=9.286, **p=0.002**
NA	24	4.8	
**pN stage**			
N0	287	57	Overall, $\chi 2$=9.296**, p=0.010**
N1	115	23	N0 v N1, $\chi 2$=0.068, p=0.794
N2	80	16	N1 v N2, $\chi 2$=4.840, **p=0.028**
NA	20	4	
**UICC stage**			
I	36	7.2	Overall, $\chi 2$=3.280, p=0.194
II	262	52.2	I v II, $\chi 2$=1.049, P=0.306
III	204	40.6	II v III, $\chi 2$=1.591, p=0.207

This cohort was retrieved from NCBI, accession number of dataset GSE39582 (https://www.ncbi.nlm.nih.gov/geo/geo2r/?acc=GSE39582), Public dataset [12]. Significant values are highlighted in bold.

Table S3. Biomarkers screened on the discovery cohort of colorectal cancer.

Biomarker	Source	Immunohistochemistry platform and scoring system	Reference
CYP4A11<br>CYP4F11<br>CYP4V2<br>CYP4Z1	Mouse monoclonal, Ximbio [M25-P2A10]<br>Mouse monoclonal, Ximbio [F21 P6 F5]<br>Mouse monoclonal, Ximbio [M29-P3B10]<br>Mouse monoclonal, Ximbio [N7-P2G5*D8]	Dako, semi quantitative scoring	[11]
CYP26A1<br>CYP26B1<br>CYP26C1<br>LRAT	Mouse monoclonal, Ximbio [F27P6A1]<br>Mouse monoclonal, Ximbio [T5P3G2*A3]<br>Mouse monoclonal, Ximbio [T6P1C7*E7]<br>Mouse monoclonal, Ximbio [M34-P1F10]	Dako, semi quantitative scoring	[13]
CYP2R1<br>CYP7B1<br>CYP8B1<br>CYP27A1<br>CYP39A1<br>CYP46A1<br>CYP51A1	Mouse monoclonal, Ximbio [M26-P6H1]<br>Mouse monoclonal, Ximbio [M17-P3F2]<br>Mouse monoclonal, Ximbio [M15-P3B7]<br>Mouse monoclonal, Ximbio [V29-P4B8*B2]<br>Mouse monoclonal, Ximbio [M30P6D6]<br>Mouse monoclonal, Ximbio [N8-P6E4*H8]<br>Mouse monoclonal, Ximbio [N6-P2H5*G8]	Dako, semi quantitative scoring	[10]
CIDEA<br>ELOVL3<br>ELOVL5<br>UCP1	Mouse monoclonal, Ximbio [V62P1E3*B10]<br>Mouse monoclonal, Ximbio [V61 P2B3*D10]<br>Mouse monoclonal, Ximbio [Z88]<br>Mouse monoclonal, Ximbio [Vab12 P4B12*A12]	Dako, semi quantitative scoring	[15]
TIAM1	Sheep polyclonal, R&D Systems (AF5038)	Dako, semi quantitative scoring	[14]
P-cofilin<br>LIMK2	Rabbit monoclonal, Cell Signaling Technology (Ser3)<br>Mouse monoclonal, Santa Cruz Biotechnology	Dako, automated quantitative scoring, Slidpath software	[18]

**Biomarker**	**Source**	**Immunohistochemistry platform and scoring system**	**Reference**
ASS1<br>OTC	Mouse monoclonal, Polaris Pharmaceuticals Inc.<br>Rabbit polyclonal, Abcam (ab91418)	Leica, automated quantitative scoring, Aperio ImageScope software	[17]
CD3<br>CD4<br>CD8<br>FOXP3<br>ICOS<br>IDO1<br>PDL1<br>CD20	Rabbit monoclonal, Ventana (2GV6)<br>Rabbit monoclonal Ventana (SP35)<br>Mouse Monoclonal, Dako (CD/144B)<br>Rabbit monoclonal, LsBio (SP97)<br>Rabbit monoclonal, Cell Signaling (D1K2T)<br>Rabbit monoclonal, Cell Signaling (D5J4E)<br>Rabbit monoclonal, Ventana (SP236)<br>Mouse monoclonal, Dako (L26)	Ventana and Leica platforms, automated quantitative scoring, QuPath image analysis	[16]
HMGB1	Rabbit monoclonal, Abcam (ab79823)	Dako, semi quantitative scoring	Unpublished
SLA2	Mouse monoclonal, Ximbio [Z30P1F12*F4]	Dako, semi quantitative scoring	Unpublished
TNFRSF18	Mouse monoclonal, Ximbio [T10P2H9*B9]	Dako, semi quantitative scoring	Unpublished
PRDM16	Rabbit polyclonal, Abcam (ab106410)	Dako, semi quantitative scoring	Unpublished
EphB6	Discontinued, Abcam, (ab56546)	Dako, semi quantitative scoring	Unpublished
SNTB2	Rabbit polyclonal, Merck (HPA001042)	Dako, semi quantitative scoring	Unpublished
CK7	Rabbit monoclonal, Ventana (SP52)	Ventanta, semi quantitative scoring	Unpublished
CK20	Rabbit monoclonal, Ventana (SP33)	Ventanta, semi quantitative scoring	Unpublished
CDX2	Rabbit monoclonal, Roche (EPR2764Y)	Ventanta, semi quantitative scoring	Unpublished
HER2	Rabbit monoclonal, Roche (4B5)	Ventanta, semi quantitative scoring	Unpublished
PTEN	Rabbit monoclonal, Cell Signaling	Dako, automated quantitative scoring, Slidpath software	Unpublished
VAV1	Rabbit monoclonal, Abcam (ab52640)		
γ-H2AX	Mouse monoclonal, Abcam (9F3/ab26350)		
p-AKT	Rabbit monoclonal, Merck (ser473)		
p21	Mouse monoclonal, Cell Signaling		

Biomarker	Source	Immunohistochemistry platform and scoring system	Reference
P53	Mouse monoclonal, Dako (DO-7)	Dako, automated quantitative scoring, Slidpath software	Unpublished
STAT1	Rabbit monoclonal, Cell Signaling Technology (D1K9Y)		
NOTCH	Rabbit monoclonal, Abcam (ab52627)	Dako, semi quantitative scoring	Unpublished
DIAPH2	Mouse monoclonal, Ximbio [V78P3C10*D3]	Dako, semi quantitative scoring	Unpublished
BAG3	Mouse monoclonal, Ximbio [V65P1E8*D2]	Dako, semi quantitative scoring	Unpublished
DRAM1	Mouse monoclonal, Ximbio [M3-P4B4]	Dako, semi quantitative scoring	Unpublished
DRAM2	Mouse monoclonal, Ximbio [VAB24-	Dako, semi quantitative scoring	Unpublished
BCLAF1	Mouse monoclonal, Ximbio [M33-P5B11]	Dako, semi quantitative scoring	Unpublished
AARS	Mouse monoclonal, Ximbio [M6-P2E5]	Dako, semi quantitative scoring	Unpublished
ERO1LB	Mouse monoclonal, Ximbio [M37-P5D11]	Dako, semi quantitative scoring	Unpublished
WSB1	Mouse monoclonal, Ximbio [K5P2H10*E10]	Dako, semi quantitative scoring	Unpublished
SPATA2	Mouse monoclonal, Ximbio [Z1P1F10*F12]	Dako, semi quantitative scoring	Unpublished
SPATA25	Mouse monoclonal, Ximbio [Z37P3H11*B2]	Dako, semi quantitative scoring	Unpublished
SPATA2L	Mouse monoclonal, Ximbio [Z2P2D5*C8]	Dako, semi quantitative scoring	Unpublished
ELOVL7	Mouse monoclonal, Ximbio [Z26]	Dako, semi quantitative scoring	Unpublished
CYP2J2	Mouse monoclonal, Ximbio [Z9P1G9*A2]	Dako, semi quantitative scoring	Unpublished
GCSP	Mouse monoclonal, Ximbio [Z44P4C6*F5]	Dako, semi quantitative scoring	Unpublished
UCP2	Mouse monoclonal, Ximbio [A1P2A9*F5]	Dako, automated quantitative scoring, QuPath image analysis	Unpublished

Table S4. Peptide sequences used as immunogens to generate monoclonal antibodies which have not been previously tested on the Grampian patient cohort.

Protein	Hybridoma clone	Peptide sequence	Peptide location
DIAPH2	V78P3C10*D3	RHNGAISSK	1093-1101
TIGAR	M2-P4H2	IINFEEGREV	239-248
BAG3	V65P1E8*D2	MTDTPGNPAAP	565-575
DRAM1	M3-P4B4	EKDYVYHVVS	192 - 201
DRAM2	VAB24-P1C8*A3	HGLTLYDTAP	242-251
BCLAF	M33-P5B11	KYQGDGIVED	900 – 909
AARS	M6-P2E5	TSFAQLRLGD	956-965
EROlLb	M37-P5D11	LAPSRGEDDG	220-229
WSB1	K5P2H10*E10	RRVMPTQEVQE	396-406
SLA2	Z30P1F12*F4	LRESLSFY	241-248
TNFRSF18	T10P2H9*B9	PWQQKWVQE	206-214
SPATA2	Z1P1F10*F12	KSTQLSHLVYR	510-520
SPATA2L	Z2P2D5*C8	LLYNSPGARP	415-424
SPATA25	Z37P3H11*B2	LVRSKRGQP	214-222
ELOVL7	Z26	RAYTKGQRLP	259-268
CYP2J2	Z9P1G9*A2	KFTFRPPNNE	468-477
GCSP	Z44P4C6*F5	PFSEQKRAS	1011-1020
UCP2	A1P2A9*F5	AACTSREAPF	300-309

Table S5. The numbers of normal and tumour tissue samples in the multi-tissue microarray.

Tissue type	Normal tissue	Tumour tissue (n=68)
Breast	4	4
Oesophagus	4	4
Stomach	4	4
Colon	4	4
Liver	4	4
Pancreas	4	4
Prostate	4	4
Kidney	4	4
Bladder tumour (urothelial cell carcinoma)	-	4
Lymph node/lymphoma	1	3
Sarcoma/GIST	-	8
Adrenal-normal	4	-
Thyroid	2	2
Ovary-tumour	-	4
Endometrium-tumour	-	4
Normal skin/melanoma	2	3
Lung	4	4
Testis	2	4
Placenta	4	-

The multi-tissue microarray was developed by the NHS Grampian Biorepository, Aberdeen, UK.

Table S6. Dilutions, antigen retrieval conditions, subcellular localisation of antibodies which have not previously been published.

Biomarker	Dilution	Antigen retrieval	Subcellular localisation
DIAPH2	Neat	Yes, pH6	Cytoplasmic
TIGAR	Neat	Yes, pH6	Cytoplasmic
BAG3	Neat	Yes, pH6	Cytoplasmic
DRAM1	Neat	Yes, pH6	Cytoplasmic
DRAM2	Neat	Yes, pH6	Cytoplasmic
BCLAF1	Neat	Yes, pH6	Cytoplasmic and nuclear
AARS	1:20	Yes, pH6	Cytoplasmic
ERO1LB	Neat	Yes, pH6	Cytoplasmic
WSB1	Neat	Yes, pH6	Cytoplasmic
SLA2	1:5	Yes, pH6	Cytoplasmic
TNFRSF18	Neat	Yes, pH6	Cytoplasmic
SPATA2	Neat	Yes, pH6	Cytoplasmic
SPATA25	Neat	Yes, pH6	Cytoplasmic and nuclear
SPATA2L	1:10	Yes, pH6	Cytoplasmic and nuclear
ELOVL7	Neat	Yes, pH6	Cytoplasmic
CYP2J2	Neat	No	Cytoplasmic
GCSP	Neat	Yes, pH6	Cytoplasmic
UCP2	Neat	Yes, pH6	Cytoplasmic
PRDM16	1:50	Yes, pH6	Cytoplasmic and nuclear
EphB6	1:50	Yes, pH6	Cytoplasmic
SNTB2	1:50	Yes, pH6	Cytoplasmic and nuclear
HMGB1	1:400	Yes, pH6	Cytoplasmic and nuclear
STAT1	1:1000	Yes, pH6	Cytoplasmic
PTEN	1:150	Yes, pH 7.8	Cytoplasmic
γ-H2AX	1:200	Yes, pH 6	Cytoplasmic
p-AKT	1:200	Yes, pH 6	Cytoplasmic
P21	1:50	Yes, pH 6	Cytoplasmic
NOTCH	1:100	Yes, pH 6	Cytoplasmic
VAV1	1:50	Yes, pH 6	Cytoplasmic

CK7, CK20, CDX2, HER2 and P53 were all stained following their established diagnostic protocols using Ventana platform.

Table S7. Comparison of the expression of each protein in normal colonic mucosa and different UICC stages in primary colorectal cancer using antibodies that have not previously been published on the discovery cohort.

Biomarker	Immunoreactivity (p-value, normal *versus* stage I)	Change in expression	Immunoreactivity (p-value, stage I *versus* stage II)	Change in expression	Immunoreactivity (p-value, stage II *versus* stage III)	Change in expression	Immunoreactivity (p-value, normal versus tumour)	Change in expression
CK7	**p=0.027**	↑	p=0.311	-	**p=0.044**	↑	P=0.281	-
CK20	na	na	**p=0.002**	↓	p=0.657	-	na	na
CDX2	na	na	p=0.195	-	**p<0.001**	↓	na	na
HER2	na	na	p=0.384	-	p=0.985	-	na	na
ELOVL7	p=0.096	-	**p=0.041**	↑	p=0.743	-	**p=0.010**	↑
PRDM16 nuclear<br>PRDM16 cytoplasmic	**p<0.001**<br>**p<0.001**	↓<br>↑	p=0.679<br>p=0.136	-	p=0.321<br>p=0.795	-<br>-	**p<0.001**<br>**p<0.001**	↓<br>↑
SLA2	**p=0.007**	↑	**p=0.004**	↑	**p=0.003**	↓	**p<0.001**	↑
WSB1	p=0.280	-	p=0.571	-	**p<0.001**	↓	p=0.061	-
CYP2J2	**p<0.001**	↑	p=0.592	-	**p<0.001**	↓	**p<0.001**	↑
P53	**p=0.003**	↓	**p=0.004**	↓	p=0.845	-	**p<0.001**	↓
p-Akt	**p=0.003**	↓	**p=0.031**	↓	**p=0.014**	↓	**p<0.001**	↓
PTEN epithelial<br>PTEN stromal	p=0.162<br>p=0.282	-<br>-	**p<0.001**<br>**p<0.001**	↓<br>↓	**p=0.036**<br>**p=0.011**	↓<br>↓	**p<0.001**<br>**p<0.001**	↓<br>↓
Notch	**p<0.001**	↓	**p=0.005**	↓	**p=0.014**	↑	**p<0.001**	↓
yH2 epithelial	p=0.101	-	p=0.545	-	p=0.682	-	**p=0.016**	↑
Vav1 epithelial<br>Vav1 stromal	**p=0.001**<br>**p<0.001**	↓<br>↓	**p=0.008**<br>p=0.362	↓<br>-	p=0.856<br>p=0.269	-<br>-	**p<0.001**<br>**p<0.001**	↓<br>↓
STAT1	p=0.064	-	**p<0.001**	↓	**p<0.001**	↓	**p<0.001**	↓
P21	**p=0.039**	↓	p=0.123	-	**p=0.001**	↓	**p<0.001**	↓
SNTB2N<br>SNTB2C	**p<0.001**<br>**p<0.001**	↓<br>↑	p=0.588<br>**p<0.001**	-<br>↓	p=0.754<br>**p=0.005**	-<br>↓	**p<0.001**<br>**p<0.001**	↓<br>↑
HMGB1 cytoplasmic<br>HMGB1 nuclear	**P=0.003**<br>**p<0.001**	↑<br>↓	**p=0.047**<br>p=0.629	↑<br>-	p=0.832<br>p=0.852	-<br>-	**p<0.001**<br>**p<0.001**	↑<br>↓
EphB6	**p<0.001**	↑	p=0.059	-	**p=0.022**	↓	**p<0.001**	↑
TIGAR	p=0.831	-	p=0.152	-	**p=0.003**	↓	p=0.768	-
AARS	p=0.242	-	p=0.366	-	p=0.175	-	p=0.120	-
DIAPH2	**p=0.028**	↑	p=0.947		**p<0.001**	↓	p=0.204	-
SPATA2L nuclear<br>SPATA2L cytoplasmic	**p<0.001**<br>p=0.515	↑<br>-	p=0.374<br>**p<0.001**	-<br>↑	p=0.232<br>p=0.874	-<br>-	**p<0.001**<br>**p<0.001**	↑<br>↑
GCSP	**p<0.001**	↑	p=0.150	-	**p<0.001**	↓	**p<0.001**	↑

BAG3	**p<0.001**	↑	**p=0.030**	↑	**p=0.012**	↓	**p<0.001**	↑
DRAM2	**p<0.001**	↑	p=0.998	-	p=0.303	-	**p<0.001**	↑
ERO1lB	**p=0.001**	↑	p=0.648	-	p=0.949	-	**p<0.001**	↑
SPATA2	**p=0.003**	↑	p=0.852	-	p=0.102	-	**p=0.001**	↑
SPATA25 nuclear	p=0.302	-	**p=0.009**	↓	p=0.241	-	**p=0.006**	↓
SPATA25 cytoplasmic	**p<0.001**	↑	p=0.117	-	p=0.726	-	**p<0.001**	↑
TNFRSF18s	**p<0.001**	↑	p=0.222	-	p=0.859	-	**p<0.001**	↑
UCP2	**p<0.001**	↑	p=0.094	-	**p=0.005**	↑	**p<0.001**	↑
BCLAF1 nuclear	p=0.127	-	p=0.884	-	**p=0.009**	↓	p=0.416	-
BCLAF1 cytoplasmic	p=0.248	-	**p=0.027**	↑	**p=0.002**	↓	p=0.107	-
DRAM1	**P=0.001**	↑	**p=0.016**	↑	**p=0.003**	↓	**p<0.001**	↑

Evaluation of normal colonic epithelium versus primary tumour samples for immunoreactivity (Mann-Whitney U test, ↑=increased in tumour, ↓=decreased in tumour, =no change between tumour and normal). Significant values are highlighted in bold.

Table S8. Starting list of biomarkers included in the biomarker combinatorial analysis.

Biomarker	Published	Missing	Optimal cut-off point	Survival association	Association with survival
p-cofilin	Published	65	Strong v rest	$\chi2$=12.749, p=0.005	**Higher expression is associated with better prognosis N=19**
LIMK2		29	Neg v weak v mod v strong	$\chi2$=25.388, p<0.001	
CIDEA		45	Negative v positive	$\chi2$=7.424, p=0.006	
ELOVL3		23	Strong v rest	$\chi2$=4.353, p=0.037	
ELOVL5		29	Low v high	$\chi2$=6.992, p=0.008	
UCP1		37	Negative v positive	$\chi2$=19.238, p<0.001	
TIAM1n		30	Low v high	$\chi2$=5.010, p=0.025	
CD3 stromal		63	Neg v weak v mod v strong	$\chi2$=39.730, p<0.001	
CD4 stromal		69	Neg v weak v mod v strong	$\chi2$=16.701, p=0.001	
CD8 stromal		81	Neg v weak v mod v strong	$\chi2$=17.435, p=0.001	
FOXP3 stromal		63	Neg v weak v mod v strong	$\chi2$=38.302, p<0.001	
ICOS stromal		79	Neg v weak v mod v strong	$\chi2$=47.770, p<0.001	
IDO1 stromal		81	Neg v weak v mod v strong	$\chi2$=10.093, p=0.001	
CDX2	Unpublished	39	Negative v positive	$\chi2$=7.970, p=0.005	
UCP2		37	Low v high	$\chi2$=5.401, p=0.02	
p-AKT		10	Low v high	$\chi2$=5.401, p=0.02	
PTEN epithelial<br>PTEN stromal		13/14	Strong v rest<br>Strong v rest	$\chi2$=13.155, p<0.001<br>$\chi2$=11.784, p=0.001	
STAT1		0	Neg v weak v mod v strong	$\chi2$=37.260, p<0.001	
SPATA2		41	Negative v positive	$\chi2$=6.285, p=0.012	
CYP4A11	Published	25	Neg v weak v mod v strong	$\chi2$=9.080, p=0.028	**Higher expression is associated with worse prognosis N=8**
CYP8B1		27	Strong v rest	$\chi2$=14.298, p<0.001	
CYP26B1		28	Strong v rest	$\chi2$=11.092, p=0.001	
CYP27A1		28	Strong v rest	$\chi2$=7.135, p=0.008	
CYP39A1		21	Negative v Positive	$\chi2$=25.144, p<0.001	
CYP46A1		25	Strong v rest	$\chi2$=8.179, p=0.004	
LRAT		28	Low v high	$\chi2$=5.039, p=0.025	
DRAM1	Unpublished	27	Negative v Positive	$\chi2$=4.243, p=0.039	
SPATA2L		20	Negative v Positive	$\chi2$=7.292, p=0.007	Excluded

Table S9. Associations of the biomarker signature and clinicopathological variables. Survival analysis of the biomarker signature was stratified by clinicopathological variables.

Variable	Subgroup	Chi-squared associations with the signature		Survival analysis	
		$\chi2$	p-value	$\chi2$	p-value
**Age**	<70	5.328	p=0.255	24.643	**p<0.001**
	≥70			52.988	**p<0.001**
**Site**	colon	10.502	p=0.232	59.879	**p<0.001**
	rectum			12.556	**p=0.014**
**MMR status**	proficient	20.518	**p<0.001**	58.634	**p<0.001**
	defective			5.378	p=0.251
**Tumour differentiation**	mod/well	10.896	**p=0.021***	65.775	**p<0.001**
	poor			4.389	p=0.356
**EMVI**	no	26.922	**p<0.001**	43.346	**p<0.001**
	yes			6.880	p=0.142
**Lymph nodes**	N0	84.349	**p<0.001**	15.660	**p=0.004**
	N1			17.819	**p=0.001**
	N2			13.898	**p=0.008**
**Tstage**	T1	40.199	**p<0.001***	1.762	p=0.623
	T2			67.516	**p<0.001**
	T3			31.371	**p<0.001**
	T4			10.575	**p=0.032**
**UICC stage**	I	73.419	**p<0.001**	15.307	**p=0.004**
	II			5.428	p=0.246
	III			21.951	**p<0.001**
**Pathological risk classification**	low risk	48.964	**p<0.001**	15.758	**p=0.003**
	high risk			24.688	**p<0.001**

Pathological risk classification: low risk (pT1-pT3 and (G1 or G2) and V0 and pN0) Vs high risk ((pN0 and pT4 or G3 or V1) or any pN1 or pN2). * Fisher exact test expected value of cells< 5). Significant values are highlighted in bold.

Table S10. Comparisons of the clinicopathological characteristics of cases for which current pathological risk classifications differ from the risk groups of the biomarker signature.

Variable	Subgroup	Low-risk pathological		High-risk pathological	
		Low-risk biomarkers	High-risk biomarkers	Low-risk biomarkers	High-risk biomarkers
**Survival**	dead	**39 (23.9%)**	**26 (50%)**	**60 (50.4%)**	**107 (74.3%)**
	alive	124 (76.1%)	26 (50%)	59 (49.6%)	37 (25.7%)
**Age**	<70	68 (42.7%)	28 (53.8%)	59 (46.9%)	67 (46.5%)
	≥70	95 (58.3%)	24 (46.2%)	60 (50.4%)	77 (50.4%)
**Site**	colon	126 (77.3%)	40 (76.9%)	92 (77.3%)	111 (77.1%)
	rectum	37 (22.7%)	12 (23.1%)	27 (22.7%)	33 (22.9%)
**MMR status**	proficient	**155 (95.1%)**	**42 (80.8%)**	**106 (89.1%)**	**109 (78.4%)**
	defective	8 (4.9%)	10 (19.2%)	13 (10.9%)	30 (21.6%)
**Tumour differentiation**	mod/well	163 (100%)	52 (100%)	107 (89.9%)	125 (86.8%)
	poor	0	0	12 (10.1%)	19 (13.2%)
**EMVI**	no	163 (100%)	52 (100%)	78 (65.5%)	78 (54.2%)
	yes	0	0	**41 (34.5%)**	**66 (45.8%)**
**Lymph nodes**	N0	163 (100%)	52 (100%)	32 (26.9%)	16 (11.1%)
	N1	0	0	53 (44.5%)	81 (56.3%)
	N2	0	0	34 (28.6%)	47 (32.6%)
**Tstage**	T1	19 (11.7%)	3 (5.8%)	3 (2.5%)	1 (0.7%)
	T2	61 (37.4%)	14 (26.9%)	11 (9.2%)	6 (4.2%)
	T3	83 (50.9%)	35 (67.3%)	76 (63.9%)	98 (68.1%)
	T4	0	0	29 (24.4%)	39 (27.1%)
**UICC stage**	I	80 (49.1%)	17 (32.7%)	2 (1.7%)	1 (0.7%)
	II	**83 (50.9%)**	**35 (67.3%)**	**30 (25.2%)**	**15 (10.4%)**
	III	0	0	87 (73.1%)	128 (88.9%)

Pathological risk classification: low risk (pT1-pT3 and (G1 or G2) and V0 and pN0) Vs high risk ((pN0 and pT4 or G3 or V1) or any pN1 or pN2).

The REMARK checklist

	Item to be reported	Page no.
**INTRODUCTION**		
1	State the marker examined, the study objectives, and any pre-specified hypotheses.	4,6,7,8 and 9 (Table S3)
**MATERIALS AND METHODS**		
Patients		
2	Describe the characteristics (e.g., disease stage or co-morbidities) of the study patients, including their source and inclusion and exclusion criteria.	6,7 and 8 (Table S1)
3	Describe treatments received and how chosen (e.g., randomised or rule-based).	6 ,7 and 8
Specimen characteristics		
4	Describe type of biological material used (including control samples) and methods of preservation and storage.	6 ,7 and 8, Materials and methods S1
Assay methods		
5	Specify the assay method used and provide (or reference) a detailed protocol, including specific reagents or kits used, quality control procedures, reproducibility assessments, quantitation methods, and scoring and reporting protocols. Specify whether and how assays were performed blinded to the study endpoint.	6, Materials and methods S1, Table S3-S6
Study design		
6	State the method of case selection, including whether prospective or retrospective and whether stratification or matching (e.g., by stage of disease or age) was used. Specify the time period from which cases were taken, the end of the follow-up period, and the median follow-up time.	6-8
7	Precisely define all clinical endpoints examined.	6
8	List all candidate variables initially examined or considered for inclusion in models.	Table S3, S7
9	Give rationale for sample size; if the study was designed to detect a specified effect size, give the target power and effect size.	NA
Statistical analysis methods		
10	Specify all statistical methods, including details of any variable selection procedures and other model-building issues, how model assumptions were verified, and how missing data were handled.	9-1
11	Clarify how marker values were handled in the analyses; if relevant, describe methods used for cutpoint determination.	9-11
**RESULTS**		
Data		
12	Describe the flow of patients through the study, including the number of patients included in each stage of the analysis (a diagram may be helpful) and reasons for dropout. Specifically, both overall and for each subgroup extensively examined report the numbers of patients and the number of events.	NA

13	Report distributions of basic demographic characteristics (at least age and sex), standard (disease-specific) prognostic variables, and tumour marker, including numbers of missing values.	Table S1
Analysis and presentation		
14	Show the relation of the marker to standard prognostic variables.	Table 1&2
15	Present univariable analyses showing the relation between the marker and outcome, with the estimated effect (e.g., hazard ratio and survival probability). Preferably provide similar analyses for all other variables being analysed. For the effect of a tumour marker on a time-to-event outcome, a Kaplan–Meier plot is recommended.	Figure 2 &3
16	For key multivariable analyses, report estimated effects (e.g., hazard ratio) with confidence intervals for the marker and, at least for the final model, all other variables in the model.	Table 1 &2
17	Among reported results, provide estimated effects with confidence intervals from an analysis in which the marker and standard prognostic variables are included, regardless of their statistical significance.	Table 1 &2
18	If done, report results of further investigations, such as checking assumptions, sensitivity analyses, and internal validation.	NA
**DISCUSSION**		
19	Interpret the results in the context of the pre-specified hypotheses and other relevant studies; include a discussion of limitations of the study.	16-21
20	Discuss implications for future research and clinical value.	16-21
